# Integration of Alzheimer’s disease genetics and myeloid genomics identifies disease risk regulatory elements and genes

**DOI:** 10.1038/s41467-021-21823-y

**Published:** 2021-03-12

**Authors:** Gloriia Novikova, Manav Kapoor, Julia TCW, Edsel M. Abud, Anastasia G. Efthymiou, Steven X. Chen, Haoxiang Cheng, John F. Fullard, Jaroslav Bendl, Yiyuan Liu, Panos Roussos, Johan LM Björkegren, Yunlong Liu, Wayne W. Poon, Ke Hao, Edoardo Marcora, Alison M. Goate

**Affiliations:** 1 Ronald M. Loeb Center for Alzheimer’s Disease, Department of Neuroscience, Icahn School of Medicine at Mount Sinai, New York, NY USA; 2 Department of Genetics and Genomic Sciences, Icahn School of Medicine at Mount Sinai, New York, NY USA; 3 Department of Neurobiology & Behavior, University of California Irvine, Irvine, CA USA; 4 Sue and Bill Gross Stem Cell Research Center, University of California Irvine, Irvine, CA USA; 5 Department of Medical and Molecular Genetics, Indiana University School of Medicine, Indianapolis, IN USA; 6 Center for Computational Biology and Bioinformatics, Indiana University School of Medicine, Indianapolis, IN USA; 7 Department of Psychiatry, Icahn School of Medicine at Mount Sinai, New York, NY USA; 8 Integrated Cardio Metabolic Centre, Department of Medicine, Karolinska Institutet, Karolinska Universitetssjukhuset, Huddinge, Sweden; 9 Institute for Memory Impairments and Neurological Disorders, University of California Irvine, Irvine, CA USA

**Keywords:** Genome informatics, Functional genomics, Alzheimer's disease, Neuroimmunology

## Abstract

Genome-wide association studies (GWAS) have identified more than 40 loci associated with Alzheimer’s disease (AD), but the causal variants, regulatory elements, genes and pathways remain largely unknown, impeding a mechanistic understanding of AD pathogenesis. Previously, we showed that AD risk alleles are enriched in myeloid-specific epigenomic annotations. Here, we show that they are specifically enriched in active enhancers of monocytes, macrophages and microglia. We integrated AD GWAS with myeloid epigenomic and transcriptomic datasets using analytical approaches to link myeloid enhancer activity to target gene expression regulation and AD risk modification. We identify AD risk enhancers and nominate candidate causal genes among their likely targets (including *AP4E1, AP4M1, APBB3, BIN1, MS4A4A, MS4A6A, PILRA, RABEP1, SPI1, TP53INP1*, and *ZYX*) in twenty loci. Fine-mapping of these enhancers nominates candidate functional variants that likely modify AD risk by regulating gene expression in myeloid cells. In the MS4A locus we identified a single candidate functional variant and validated it in human induced pluripotent stem cell (hiPSC)-derived microglia and brain. Taken together, this study integrates AD GWAS with multiple myeloid genomic datasets to investigate the mechanisms of AD risk alleles and nominates candidate functional variants, regulatory elements and genes that likely modulate disease susceptibility.

## Abstract (web summary)

This study integrates Alzheimer’s disease (AD) GWAS data with myeloid cell genomics, and reports that myeloid active enhancers are most burdened by AD risk alleles. The authors also nominate candidate causal regulatory elements, variants and genes that likely modulate the risk for AD.

## Introduction

Alzheimer’s disease (AD) is the most common type of dementia with a global burden of approximately 50 million people and no disease-modifying treatments available^1^. Several lines of genetic evidence implicate myeloid cells in the etiology of AD^2^. Whole-exome sequencing and microarray studies have identified rare coding variants associated with AD in genes (e.g., *TREM2*^3^*, SORL1*^4^*, ABI3*^5^*, PLCG2*^5^ and *ABCA7*^6^) that play important roles in myeloid cells of the brain (microglia) and peripheral tissues (e.g., monocytes and macrophages) and have high relative expression levels in microglia compared to other brain cell types^7^. Genome-wide association studies (GWAS) have identified common non-coding variants associated with AD in more than 40 loci^8^, but the identification of the functional variants and causal genes underlying these statistical associations has been lacking. Earlier studies have focused on mapping candidate causal genes to AD risk loci using whole-blood and brain expression quantitative trait loci (eQTL) datasets^9–11^. However, using tissue-level data poses obstacles to identifying myeloid-specific signals, because myeloid cells (microglia and monocytes) represent small fractions (~10%) of the total cell population in their respective tissues (brain and peripheral blood). More importantly, given the strong enrichment of AD risk alleles in myeloid-specific epigenomic annotations and expressed genes^12,13^, it is imperative to investigate their impact on myeloid epigenomes and transcriptomes in the modulation of AD susceptibility.

Here, we show that AD risk alleles are specifically enriched in active enhancers of monocytes, macrophages and microglia and identify transcription factor binding motifs (TFBMs) overrepresented within these regulatory elements. We further identify myeloid transcription factors (TFs) whose binding sites at active enhancers are likely burdened by AD risk variants. Given the selective enrichment of AD risk alleles in myeloid active enhancers, we sought to link the activity of myeloid enhancers that contain AD risk variants to target gene expression regulation and AD risk modification. To accomplish this we use two complementary approaches. First, we map myeloid active enhancers that contain AD risk alleles (AD risk enhancers) to their target genes by integrating chromatin interactions (promoter-capture Hi–C) and eQTL datasets from monocytes and macrophages. This approach allows us to nominate candidate causal genes in eleven genome-wide significant and five suggestive AD risk loci, including *TP53INP1, APBB3, RABEP1,* and *SPPL2A*. In our second approach, we use Summary data-based Mendelian Randomization (SMR)^14^ to investigate the causal relationship between chromatin activity, target gene expression, and AD risk modification. This approach allows us to identify specific active chromatin regions that likely modify AD risk by regulating the expression of one or more of their target genes in 12 loci. Importantly, the target genes of the myeloid active enhancers identified by these two analytical approaches are highly consistent and implicate the endolysosomal system of myeloid cells in the etiology of AD. We further fine-map AD risk enhancers to identify candidate functional variants that likely affect TF binding and regulate gene expression in seven loci, and validate one of these variants in the MS4A locus in human induced pluripotent stem cell (hiPSC)-derived microglia and brain.

## Results

### AD risk alleles are specifically enriched in active enhancers of monocytes, macrophages, and microglia

**Fig. 1:**
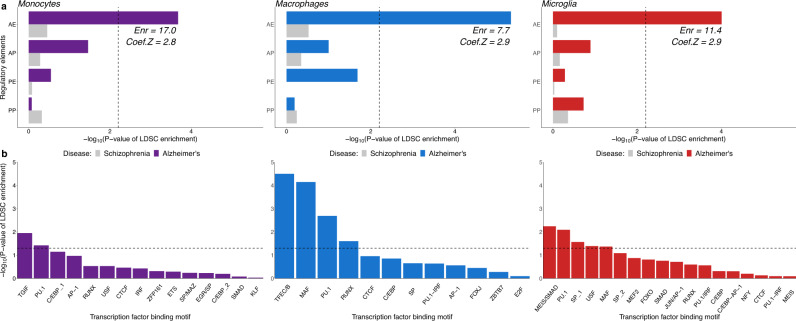
AD risk alleles are specifically enriched in myeloid active enhancers and in putative transcription factor binding sites located in these enhancers. **a** -Log10 of enrichment *P*-values obtained from stratified LD Score Regression (LDSC) analysis of AD SNP heritability partitioned by active enhancer (AE), active promoter (AP), primed enhancer (PE) and primed promoter (PP) annotations in monocytes, macrophages, and microglia. Enr = Enrichment of AD SNP heritability partitioned by active enhancer annotations. Dashed line indicates the Bonferroni-corrected significance threshold. The enrichment standard errors for active enhancers are 3.8, 1.3, and 2.6 for monocytes, macrophages, and microglia, respectively. **b** -Log10 of enrichment *P*-values obtained from stratified LD Score Regression (LDSC) analysis of AD SNP heritability partitioned by ATAC-Seq subsets. The subsets were obtained by stratifying ATAC-Seq regions in monocytes, macrophages, and microglia by the presence of the binding motif of TFs (listed on the x-axis) that were found to be overrepresented in active myeloid enhancers and expressed in monocytes, macrophages, and microglia, respectively (TPM ≥ 1)^15^. Dashed line indicates the nominal significance threshold.

Our earlier analyses showed a significant enrichment of AD risk alleles in several myeloid-specific epigenomic annotations, but not in brain or other tissues/cell types (with the exception of liver and B-lymphoid cells)^12^. To further dissect this enrichment, we used ChIP-Seq profiles of histone modifications that define the chromatin signatures of regulatory elements (H3K27ac for active enhancers and promoters, H3K4me1 for enhancers, and H3K4me2 for enhancers and promoters and H3K4me3 for promoters) from monocytes, macrophages, and microglia to annotate the genome with myeloid active enhancers (AE), active promoters (AP), primed enhancers (PE) and primed promoters (PP) (see Methods)^15^. We identified 37246, 48242, and 34014 active enhancers, 7871, 13979, and 8284 active promoters, 11534, 34623, and 52360 primed enhancers and 3107, 4028, and 3112 primed promoters in monocytes, macrophages, and microglia, respectively. To identify which of these myeloid regulatory elements are enriched for AD risk alleles, we performed stratified LD score regression (LDSC)^16^ of AD single nucleotide polymorphism (SNP) heritability partitioned by the aforementioned epigenomic annotations using the International Genomics of Alzheimer’s Project (IGAP) AD GWAS dataset^17^. This analysis revealed selective enrichment of AD risk alleles in active enhancers of monocytes, macrophages, and microglia (Fig. 1a). In contrast, schizophrenia SNP heritability (using the Psychiatric Genomics Consortium SCZ GWAS dataset as control^18^) was not enriched in any of these myeloid regulatory elements (Fig. 1a).

To identify TFs that likely regulate the activity of myeloid enhancers, we performed de novo motif analysis^19^ in open chromatin regions (identified by ATAC-Seq) that overlap with active enhancers in all three cell types (Supplementary Data 1). The binding motif for PU.1 (a transcription factor critical for myeloid and B-lymphoid cell development and function and an AD risk gene (*SPI1*)^12^) was the best match for the most highly overrepresented sequence motif in active enhancers across all three cell types, followed by AP-1, C/EBP, CTCF, and RUNX binding motifs. The binding motif for MEF2 family TFs (which includes *MEF2C* in another AD risk locus^17^) was the best match for highly overrepresented sequence motif in active enhancers of human microglia, consistent with findings in mouse microglia^20^. To test whether the binding sites of TFs that likely regulate active myeloid enhancers are enriched for AD risk variants, we stratified ATAC-Seq regions in all three cell types by the presence of the binding motifs of the TFs that were found to be overrepresented in active myeloid enhancers and expressed in monocytes, macrophages and microglia (TPM ≥ 1), and applied LDSC to quantify the enrichment of AD SNP heritability partitioned by these subsets of ATAC-Seq regions (Fig. 1b). ATAC-Seq regions overlapping with active enhancers that were positive for the PU.1 binding motif in all three cell types were enriched for AD risk alleles. MAF binding motif-positive ATAC-Seq regions were enriched for AD risk alleles in macrophage and microglial active enhancers. SMAD, USF, and SP binding motif-positive ATAC-Seq regions were enriched for AD risk alleles only in microglial active enhancers. Interestingly, a study comparing two mouse strains reported that genetic variants in Mafb, Smad3, and Usf1 binding sites affected PU.1 binding specifically in microglia, suggesting that these TFs could be binding partners of PU.1 in microglia^21^. These results show that AD risk alleles are specifically enriched in active enhancers of monocytes, macrophages, and microglia, and nominate shared and cell-type specific TFs that likely regulate the activity of these regulatory elements. Additionally, these results implicate TFs whose binding to myeloid active enhancers is likely to be affected by AD risk alleles. These results support our hypothesis that TF binding sites might be altered by AD risk variants to affect myeloid enhancer activity and gene expression, which in turn modulate disease susceptibility by altering the biology of myeloid cells.

### Integration of AD GWAS signals with myeloid epigenomic annotations, chromatin interactions (promoter-capture Hi–C), and eQTL datasets identifies candidate causal genes in sixteen AD risk loci

Promoter-enhancer interactions constitute one of the most fundamental mechanisms of gene expression regulation, where enhancer elements are brought into close proximity to cognate promoters to stimulate transcription of their target genes^19^. Given the observed enrichment of AD risk alleles in myeloid active enhancers, we reasoned that harnessing information about the spatial organization of chromatin and integrating it with epigenomic annotations and eQTLs in myeloid cells would facilitate the identification of candidate causal genes regulated by these elements in AD risk loci.

Chromatin interactions and eQTL datasets are currently not available for human microglia. However, our partitioned AD SNP heritability estimates suggest that active enhancers are enriched in monocytes, macrophages, and microglia to a similar extent, hence we used datasets from human peripheral blood monocytes and monocyte-derived macrophages as we did previously^12^. We first identified active enhancers in monocytes and macrophages that contain AD risk alleles (P ≤ 1 × 10^−6^, hereafter referred to as AD risk enhancers). Among these we then selected those that interact with at least one gene promoter and contain AD risk variants that are eQTLs for the same gene in monocytes and macrophages (FDR ≤ 5%) using the Javierre et al.^19^ promoter-capture Hi–C dataset and the Cardiogenics^22^, Fairfax et al. 2014^23^ and STARNET^24^ eQTL datasets. These analyses were performed within a single cell type: monocyte epigenomic marks were integrated with monocyte promoter capture Hi–C and 2 independent monocyte eQTL datasets (Cardiogenics and Fairfax). Similarly, macrophage epigenomic annotations were integrated with macrophage promoter capture Hi–C and 2 independent macrophage eQTL datasets (Cardiogenics and STARNET). Using this approach we nominate candidate causal genes in sixteen genome-wide significant and suggestive AD risk loci (Table 1). In some loci, this analysis identified genes that have known AD-associated coding variants (*ABCA7*^25^) and genes that have been identified as most likely causal in previous studies (*BIN1*^26^ and *PTK2B*^27^). In other loci, we uncovered co-regulation of the expression of multiple target genes by shared AD risk enhancers. For example, in the *SPI1* locus, we identified AD risk enhancers shared by *ACP2, MADD, MYBPC3, NR1H3, NUP160, PSMC3,* and *SPI1* in monocytes, and by *NUP160*, *MYBPC3,* and *SPI1* in macrophages. Similarly, in the *PILRA* locus (previously *ZCWPW1*), we identified AD risk enhancers shared by *AP4M1, PILRA, PILRB*, and *ZCWPW1* in monocytes, and by *AP4M1, MCM7, PILRA, PILRB, PVRIG,* and *STAG3* in macrophages. This could reflect the presence of either multiple causal genes at these loci or a single causal gene and several risk-neutral genes that show association by virtue of expression co-regulation. Additional evidence is necessary to distinguish between these two possibilities and prioritize one or more genes in the locus as we have shown for *SPI1* at the respective (previously *CELF1*) locus^12^.

**Table 1:** Candidate causal genes identified through integration of AD GWAS signals with myeloid active enhancer annotations, promoter-capture Hi–C, and eQTLs datasets.

Locus	Monocytes	Monocyte-derived macrophages
*BIN1*	***BIN1***	***BIN1***
*SPI1* (previously *CELF1*)	*ACP2, FNBP4*, ***MADD***, ***MYBPC3***, *MTCH2*, *NR1H3*, ***NUP160***, *PSMC3*, ***SPI1***	*CELF1*, *MTCH2*, ***MYBPC3***, ***NUP160***, *PSMC3*, ***SPI1***
*ZYX* (previously *EPHA1*)	***ZYX***	CLCN1*, EPHA1*, *FAM131B*, TAS2R41*, TAS2R60*, ***ZYX***
*MS4A*	***MS4A6A***, MS4A6E*	***MS4A6A***
*PILRA* (previously *ZCWPW1)*	***AP4M1***, *GATS*, ***PILRA***, *PILRB*, ***ZKSCAN1***, *ZCWPW1*	***AP4M1***, *GATS*, *LAMTOR4*, *MCM7*, ***PILRA***, *PILRB*, ***PVRIG***, ***STAG3***, *TRIM4*, ***ZKSCAN1***
*TP53INP1* (previously *NDUFAF6*)	*CCNE2*, ***INTS8***, ***TP53INP1***	*INTS8*, *NDUFAF6*, ***TP53INP1***
*SPPL2A*	—	*AP4E1*, *TRPM7*
*RIN3* (previously *SLC24A4*)	*RIN3*	—
*ABCA7*	*ABCA7,CNN2,CIRBP*	*ABCA7*, *CNN2*, *GPX4*
*APBB3* (previously *HBEGF*)	—	***APBB3***, ***PFDN1***
*RABEP1* (previously *SCIMP*)	***NUP88***, ***RABEP1***	***CHRNE***, ***NUP88***, ***RABEP1***
*PTK2B*	***PTK2B***	***PTK2B***
*CD55* (previously *CR1*)	—	*CD55*
*CASS4*	*AURKA*	—
*TREM2*	*NFYA*	TREML2*
*PICALM*	—	CCDC83*

Genes highlighted in bold show a significant association between gene expression and disease risk.

*Not expressed in microglia.

**Fig. 2:**
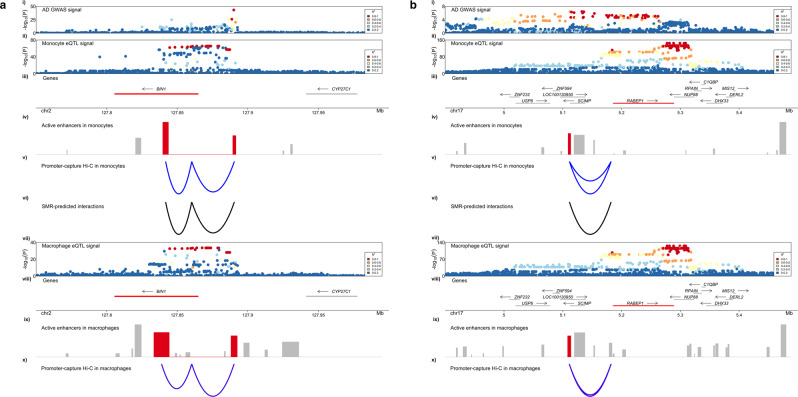
AD risk enhancers spatially interact with the promoters of *BIN1* and *RABEP1* and regulate their expression in myeloid cells. **a**. (i) AD GWAS association signal in the *BIN1* locus. (ii) eQTL signal for *BIN1* in monocytes obtained from the Cardiogenics study. (iii) Genes that reside in the locus are plotted. Likely target genes of the highlighted AD risk enhancers are shown in red. The arrow indicates the direction of transcription, while the bar indicates the gene body. (iv) Active enhancers in monocytes are plotted. The height of the bar is proportional to the strength of the epigenomic signal. AD risk enhancers that are prioritized through both Hi–C and SMR approaches are highlighted in red. (v) Promoter-capture Hi–C interactions between the *BIN1* promoter and the highlighted AD risk enhancers in monocytes. The depth of the arc is proportional to the strength of the interaction. (vi) AD risk enhancer-target gene interactions predicted by SMR analysis of causal associations between chromatin activity and *BIN1* expression in monocytes. The depth of the arc is proportional to the strength of the association. (vii) eQTL signal for *BIN1* in macrophages obtained from the Cardiogenics study. (viii) Genes that reside in the locus are plotted. Likely target genes of the highlighted AD risk enhancers are shown in red. The arrow indicates the direction of transcription, while the bar indicates the gene body. (ix) Active enhancer elements in macrophages are plotted. AD risk enhancers that interact with the gene promoter are highlighted in red. (x) Promoter-capture Hi–C interactions between the *BIN1* promoter and the highlighted AD risk enhancers in macrophages. The depth of the arc is proportional to the strength of the interaction. Both Hi–C and SMR-predicted interactions are anchored at the AD risk enhancer highlighted. **b**. (i) AD GWAS association signal in the *RABEP1* locus. (ii) eQTL signal for *RABEP1* in monocytes obtained from the Cardiogenics study. (iii) Genes that reside in the locus are plotted. Likely target genes of the highlighted AD risk enhancers are shown in red. The arrow indicates the direction of transcription, while the bar indicates the gene body. (iv) Active enhancers in monocytes are plotted. The height of the bar is proportional to the strength of the epigenomic signal. AD risk enhancers that are prioritized through both Hi–C and SMR approaches are highlighted in red. (v) Promoter-capture Hi–C interactions between the *RABEP1* promoter and the highlighted AD risk enhancers in monocytes. The depth of the arc is proportional to the strength of the interaction. (vi) AD risk enhancer-target gene interactions predicted by SMR analysis of causal associations between chromatin activity and *RABEP1* expression in monocytes. The depth of the arc is proportional to the strength of the association. vii) eQTL signal for *RABEP1* in macrophages obtained from the Cardiogenics study. (viii) Genes that reside in the locus are plotted.Target genes of the highlighted AD risk enhancers are shown in red. The arrow indicates the direction of transcription, while the bar indicates the gene body. (ix) Active enhancer elements in macrophages are plotted. AD risk enhancers that interact with the gene promoter are highlighted in red. (x) Promoter-capture Hi–C interactions between the *RABEP1* promoter and the highlighted AD risk enhancers in macrophages. The depth of the arc is proportional to the strength of the interaction. Hi–C and SMR-predicted interactions are anchored at the AD risk enhancers highlighted.

Additionally, these analyses revealed regulatory landscapes that are shared across myeloid cell types or are cell type-specific. In the *BIN1* locus, we observed conserved AD risk enhancer-promoter chromatin interactions and similar eQTL signal profiles in monocytes and macrophages, suggesting that the AD risk regulome is similar in these two cell types and points to *BIN1* as the strongest candidate causal gene at this locus (Fig. 2a). Conversely, in the *ZYX* (previously *EPHA1*) locus, we observed stronger chromatin interactions with a *ZYX* promoter in macrophages (mean interaction score 3.3 and 7.0 in monocytes and macrophages, respectively) and different eQTL signal profiles between monocytes and macrophages, suggesting that the AD risk regulome is different in these two cell types albeit pointing to the same candidate causal gene (Supplementary Figure 1). Finally, we identified candidate causal genes, such as *RABEP1* (Fig. 2b), *TP53INP1* (Supplementary Figure 2) and *APBB3* in suggestive loci. We also found that many of the genes prioritized through Hi–C in monocytes and macrophages are also associated with disease risk (SMR), including *ZYX, PILRA, AP4M1, RABEP1, APBB3* and *TP53INP1* (Table 1). In summary, this analytical approach allowed us to nominate candidate causal genes in sixteen AD risk loci.

### Integration of AD GWAS signals with myeloid epigenomic annotations, chromatin activity (hQTL) and eQTL datasets identifies candidate causal genes in twelve AD risk loci

**Fig. 3:**
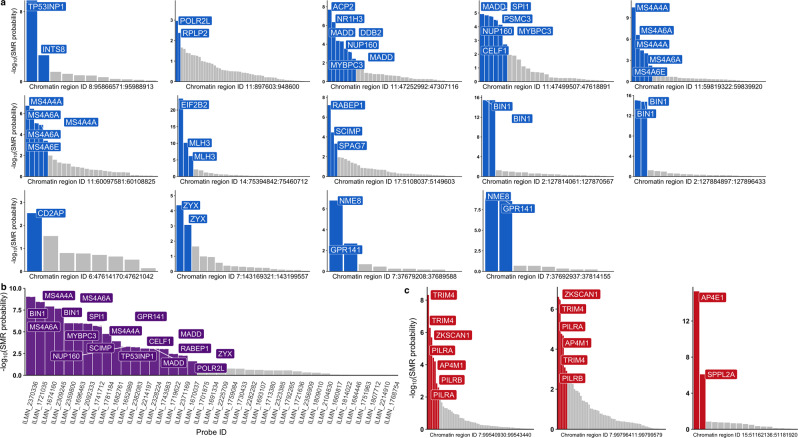
Putative causal associations between chromatin activity, target gene expression regulation and AD risk modification point to candidate causal genes in myeloid cells. **a** -Log10 of causal association probabilities between chromatin activity and gene expression in monocytes obtained through SMR analysis for each probe are plotted for each chromatin region. Probes (labeled by the respective gene) in blue indicate significant associations, while grey bars indicate non-significant associations based on a 5% FDR threshold. **b** -Log10 of causal association probabilities between gene expression and AD risk. Probes (labeled by their respective gene) in purple indicate significant associations, while grey bars indicate non-significant associations based on a 5% FDR threshold. **c** -Log10 of causal association probabilities between activity of two active chromatin regions in the PILRA locus and one active chromatin region in the SPPL2A locus and gene expression in monocytes obtained through SMR analysis for each probe are plotted. Probes (labeled by the respective gene) in red indicate significant associations, while grey bars indicate non-significant associations based on a 5% FDR threshold.

Although chromatin interactions between active enhancers and gene promoters may suggest target gene expression regulation, inferring causal relationships between chromatin activity at enhancer elements and target gene expression can provide additional evidence for such regulation and help identify genetic variants that mediate these relationships to modulate disease susceptibility. We used SMR to explore the causal path that links chromatin activity to target gene expression and AD risk modification. To accomplish this, we used datasets from monocytes^28^, since chromatin activity QTLs (hQTLs) are currently not available for human microglia or other macrophages. We first identified chromatin regions that contain AD risk alleles and overlap an active enhancer and used coloc^29^ to select those with evidence of independent or colocalized AD GWAS and hQTL signals (PP.H3.abf + PP.H4.abf ≥ 0.8) (Supplementary Data 2). To investigate the link between chromatin activity and target gene expression regulation, we used SMR to test for causal association between hQTL and eQTL effects in monocytes at the 26 regions selected using coloc as described above. We identified multiple genes that are likely regulated by the active enhancers in these regions (Fig. 3a, Table 2, Supplementary Data 3), including *BIN1, CD2AP, GPR141, MS4A4A, MS4A6A, RABEP1, SPI1, TP53INP1* and *ZYX*. We then used SMR to test for causal association between the expression of these genes and disease susceptibility. These analyses revealed specific active chromatin regions in monocytes, whose activity is causally associated with expression of their target genes, which in turn is causally associated with AD risk, including *BIN1, GPR141, MS4A4A, MS4A6A, RABEP1, SPI1, TP53INP1*, and *ZYX* (Fig. 3b, Supplementary Data 4). Seventeen of twenty-six genes nominated through causal associations between chromatin activity and gene expression and eight of fourteen genes nominated through causal associations between gene expression and disease susceptibility identified using the Cardiogenics monocyte eQTL dataset were replicated using the Fairfax monocyte eQTL dataset (Supplementary Data 5-6). Since the replication cohort is smaller, we expect that a larger number of associations would replicate in a larger cohort, given the fact that almost all genes found through associations using the Fairfax dataset were significant in the main analysis using the Cardiogenics dataset. Additionally, in *MS4A*, *SPI1, TP53INP1* and *ZYX* loci both computational approaches pointed to the same candidate causal genes (albeit nominating different enhancers), while in *BIN1* and *RABEP1* loci both approaches pointed to the same AD risk enhancers and target gene (Fig. 2a). Hence, these results provide converging evidence for target gene expression regulation by active enhancers in these regions.

**Table 2:** Candidate causal genes identified through integration of AD GWAS signals with myeloid active enhancer annotations, hQTL, and eQTL datasets.

Locus	Genes implicated through enhancer activity to gene expression associations	Genes implicated through enhancer activity to gene expression to disease risk associations
*BIN1*	*BIN1*	*BIN1*
*SPI1*	*ACP2*, *CELF1*, *DDB2*, *MADD*, *MYBPC3*, *NR1H3*, *NUP160*, *PSMC3*	*CELF1*, *MADD*, *MYBPC3*, *NUP160*, *SPI1*
*CD2AP*	*CD2AP*	—
*ZYX* (previously *EPHA1*)	*ZYX*	*ZYX*
*GPR141* (previously *NME8*)	*NME8*, *GPR141*	*GPR141*
*TP53INP1*	*INTS8*, *TP53INP1*	*INTS8*, *TP53INP1*
*MS4A*	*MS4A4A*, *MS4A6A*, MS4A6E*	*MS4A4A*, *MS4A6A*
*RABEP1* (previously *SCIMP*)	*NUP88*, *RABEP1*, *SCIMP*, *SPAG7*	*NUP88*, *RABEP1*, *SCIMP*
*EIF2B2*	*ACYP1*, *EIF2B2*, *MLH3*	—
*POLR2L*	*POLR2L*, *RPLP2*	*POLR2L*
*PILRA* ^*a*^ (previously *ZCWPW1*)	*AP4M1*, *TRIM4*, *PILRA*, *PILRB*, *ZKSCAN1*	*AP4M1*, *PILRA*, *ZKSCAN1*
*SPPL2A* ^*a*^	*AP4E1*, *SPPL2A*	*SPPL2A*

^a^Association reported with a distal enhancer that does not contain AD risk variants, but whose hQTLs colocalize with AD risk alleles.

^*^Not expressed in microglia.

Although we observed a global enrichment of AD risk alleles in myeloid active enhancers across the human genome (Fig. 1a), we discovered a small subset of loci where the regulatory elements associated with causal gene expression regulation are either not active enhancers and/or do not themselves contain AD risk alleles. For example, we identified multiple primed enhancers in monocytes that do not contain AD risk alleles but whose hQTLs are causally associated with expression of *PILRA, AP4M1* and *ZKSCAN1*, which is in turn causally associated with AD risk (Fig. 3c). Moreover, we identified an active enhancer element whose activity is regulated by AD risk alleles located at a distance from it and which is strongly associated with expression of *AP4E1* and *SPPL2A* in monocytes (Fig. 3c). In turn, expression of *SPPL2A* is causally associated with AD risk. Furthermore, this chromatin region interacts with the promoter of *SPPL2A*, providing converging evidence for regulation of *SPPL2A* expression by this regulatory element. Therefore, it is possible that AD risk alleles indirectly affect the activity of this regulatory element by functional coupling through chromatin looping or another mechanism. In summary, this analytical approach allowed us to nominate candidate causal genes in twelve AD risk loci.

### Fine-mapping using myeloid epigenomic annotations identifies candidate causal variants in seven AD risk loci

To prioritize candidate causal variants in myeloid enhancers we selected loci where we discovered significant associations between chromatin activity, gene expression and AD risk (i.e. *BIN1, GPR141, MS4A, PILRA, RABEP1, SPI1, SPPL2A, TP53INP1*, and *ZYX*). We first selected variants in high to moderate LD (R^2^ ≥ 0.8) with the tagging variant in each locus and queried them in Haploreg^30^ to identify coding variants. We identified a missense variant (rs1859788-G) in *PILRA* that is in high LD with the tagging variant (R^2^ = 0.86, Alzheimer’s Disease Genetics Consortium case-control cohort (ADGC) reference panel was used to compute LD as described previously)^12^ and was previously shown to alter the ligand binding affinity of PILRA^31^. Conditioning on this variant eliminates the AD GWAS signal at this locus (Supplementary Figure 3). The other eight AD risk loci did not contain coding variants in high LD with the tagging variant, prompting us to proceed with fine-mapping to prioritize candidate non-coding functional variants. To accomplish this we used PAINTOR, a Bayesian fine-mapping method that allows for integration of epigenomic annotations^32^. Due to the inflation of posterior probabilities when individuals in GWAS and LD reference panel are not well matched^33^, we used GWAS and LD statistics calculated using the ADGC cohort^12^. Although this approach reduces the number of loci that can be statistically fine-mapped due to the smaller sample size in ADGC, the results are more stable. We obtained and reprocessed 38 myeloid epigenomic annotations^15,34–38^, selected the ones that overlapped with active enhancers in myeloid cells and quantified their enrichment in each locus (Supplementary Figure 4). We then used PAINTOR with significantly enriched annotations (see Methods) to prioritize candidate causal variants and selected those with posterior probabilities of at least 0.1. To probe the likely effects of these variants on transcription factor binding, we screened for disruption or creation of binding motifs for TFs expressed in monocytes, macrophage and/or human microglia (TPM ≥ 1)^15^ using motifbreakR^39^.

We identified candidate non-coding functional variants in the *BIN1, MS4A* and *ZYX* loci and proposed their likely mechanism of action (Supplementary Data 7). Additionally, we employed an alternative strategy for fine-mapping for the aforementioned loci and the loci that were not significant in the ADGC GWAS (but were significant in the IGAP GWAS). Briefly, using a block partitioning algorithm^40^, conditional analyses^41^ and motif disruption/creation analyses^39^ as well as integration of active enhancer annotations and eQTL datasets in monocytes and macrophages we were able to prioritize variants with regulatory potential in seven AD risk loci (Supplementary Data 7, see Methods). As an example, in the *BIN1* locus we identified two independent AD GWAS signals. One of these signals is associated with rs6733839-T that is an eQTL for *BIN1* in human microglia^42^, resides in a PU.1 binding site in microglia and creates a binding motif for the MEF2 transcription factor, likely acting as a binding partner for PU.1 at that site. The other variant (rs13025717-T) also resides in a PU.1 binding site, is an eQTL for *BIN1* in monocytes and a binding QTL for PU.1 in a B-lymphoblastoid cell line (GM12878). This variant likely affects PU.1 binding by disrupting motifs of its binding partners, such as SP1 and KLF4^43,44^. Both of these variants demonstrated a significant difference in open chromatin accessibility in the brain between homozygotes for reference and alternative alleles, suggesting functional impact of these variants on the microglial epigenome (Supplementary Figure 6). Our findings in this locus are also supported by a recent study that nominated both rs6733839 and rs13025717 as candidate causal variants in the *BIN1* locus through integration of single-cell epigenomics and a machine learning approach for variant effect prediction^45^. Another recent preprint provided more promising independently derived data that demonstrated a significant allelic imbalance at rs6733839 in iPSC-derived macrophages, further supporting its functional impact on the myeloid epigenome^42^. Additionally, the microglial enhancer that harbors rs6733839 has been recently validated in the BIN1 locus, where a CRISPR knockout of this regulatory region leads to a microglia-specific reduction in BIN1 gene and protein expression^46^. We performed conditional analyses using candidate functional variants as covariates and confirmed that they do indeed tag the majority of AD GWAS signal in their respective loci (Supplementary Figure 5). SNP-targeted SMR analyses also confirmed that the prioritized candidate functional variants drive the association between gene expression levels in myeloid cells and AD risk in their respective loci (Supplementary Data 8).

### A candidate causal variant in the MS4A locus disrupts an anchor CTCF binding site and is associated with reduced chromatin accessibility and increased MS4A6A gene expression in myeloid cells

**Fig. 4:**
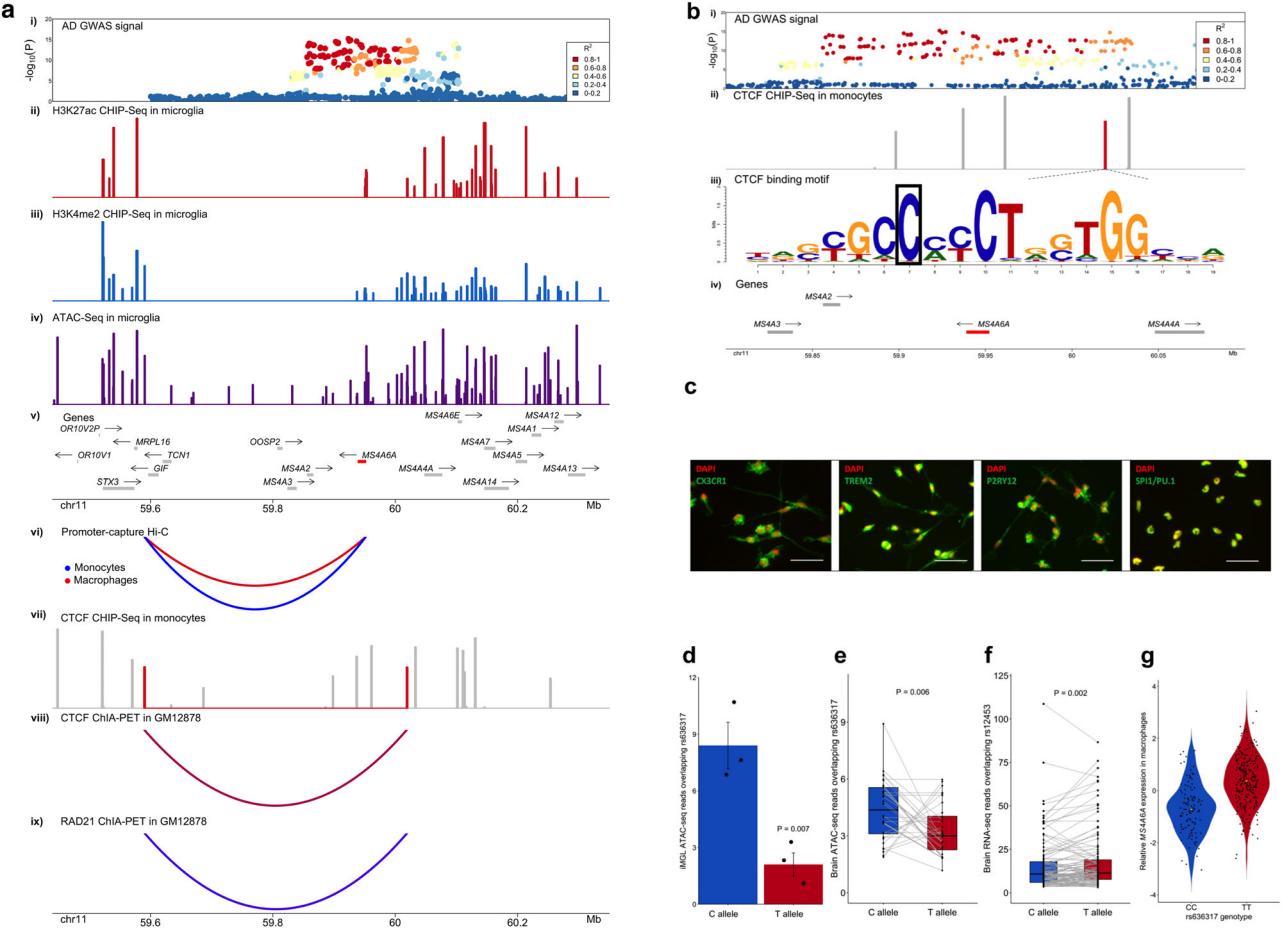
A candidate causal variant in the *MS4A* locus disrupts an anchor CTCF binding site and is associated with reduced chromatin accessibility and increased *MS4A6A* gene expression in myeloid cells and in the brain. **a** (i) AD GWAS signal in the MS4A locus. (ii) H3K27ac peaks in microglia. (iii) H3K4me2 peaks in microglia. (iv) ATAC-seq peaks in microglia. (v) Genes that reside in the locus are plotted. Putative AD risk genes are highlighted in red. The arrow indicates the direction of transcription, while the bar indicates the gene body. (vi) Strongest promoter-capture Hi–C interactions between the MS4A6A promoter and distal regulatory elements contained within the CTCF loop in monocytes (blue) and macrophages (red). (vii) CTCF ChIP-Seq peaks in monocytes. The peaks highlighted in red are anchor CTCF binding sites for the chromatin loop. (viii) CTCF ChIA-PET interactions in GM12878. (ix) RAD21 ChiA-PET interaction in GM12878. **b** (i) AD GWAS signal in the MS4A locus. (ii) CTCF ChIP-Seq peaks in monocytes. The peak highlighted in red is an anchor CTCF binding site for a chromatin loop and contains the candidate causal variant (rs636317-T). (iii) A CTCF binding motif resides in the CTCF ChIP peak highlighted in red in (ii). The candidate causal variant (rs636317-T) resides in position 7 (boxed) of this motif and is predicted to disrupt CTCF binding. (iv) Genes that reside in the locus are plotted. Putative AD risk genes are highlighted in red. The arrow indicates the direction of transcription, while the bar indicates the gene body. **c** Immunofluorescent images of microglial markers (CX3CR1, TREM2, P2RY12 and PU.1) confirming differentiation of hiPSC-derived microglia. Scale bar = 100μm. **d** Allelic imbalance of chromatin accessibility at the rs636317 site is observed in hiPSC-derived microglia. Mean normalized ATAC-Seq read counts are plotted for the protective (C) and risk-increasing (T) alleles; the dots represent each individual, centers for the error bars represent mean normalized ATAC-seq read counts and error bars represent standard errors. The protective allele (C) shows significantly more ATAC-Seq read counts than the risk-increasing allele (T) (*P*-value = 0.007, paired one-sided t-test), which is consistent with the hypothesis that the presence of the rs636317 AD risk-increasing allele leads to disruption of CTCF binding. **e** Allelic imbalance of chromatin accessibility at the rs636317 site is observed in the brain. Each pair of dots connected by a grey line represent an individual. The protective allele (C) shows significantly more ATAC-Seq read counts than the risk-increasing allele (T) (*P*-value = 0.006, paired one-sided t-test, *n* = 32), which replicates our observations in hiPSC-derived microglia. **f** Allelic imbalance in normalized brain RNA-seq reads at rs12453 site. Each pair of dots connected by a grey line represent an individual. The protective allele (C) shows significantly less MS4A6A RNA-Seq read counts than the risk-increasing allele (T) (*P*-value = 0.002, paired one-sided t-test, *n* = 118), which is consistent with our hypothesis. **g** Relative expression of MS4A6A in macrophages increases in a rs636317-T allele dose-dependent manner. Each dot represents the relative expression level of MS4A6A in each individual, while the yellow dot represents the median. Horizontal lines in box plots depict 25%, 50%, and 75% quantiles; lower whisker = lower hinge - 1.5*IQR; upper whisker = upper hinge + 1.5*IQR.

One of the prioritized candidate causal variants in the MS4A locus, the rs636317-T AD risk-increasing allele (11:60019150:C:T in GRCh37.p13 coordinates), resides in a CTCF binding site (Fig. 4b (ii)). CTCF binding sites serve as anchors for long-range chromatin loops and this protein plays a pivotal role in determining the spatial organization of chromatin to regulate gene expression^47^. The CTCF motif is highly evolutionarily conserved, and previous studies have shown that single point mutations in this motif can lead to a dramatic reduction of CTCF binding and chromatin accessibility at the site as well as alteration of chromatin looping and activity^47^. We further confirmed that rs636317-T not only resides in a CTCF ChIP-Seq peak in monocytes, but also breaks the CTCF binding consensus sequence (Fig. 4b (iii) and is a binding QTL for CTCF in a B-lymphoblastoid cell line (GM12878). Additionally, the CTCF binding QTL signal in GM12878^48^ has a 97.6% probability of colocalization with AD risk alleles at this locus. rs636317-T is a strong eQTL for *MS4A6A* in monocytes and macrophages, and the risk increasing T allele is associated with increased *MS4A6A* expression (Fig. 4g). Given that rs636317-T is predicted to disrupt a CTCF binding site, a likely scenario is that this SNP may destroy one of the two anchor CTCF binding sites in a chromatin loop, leading to altered chromatin architecture and activity in the locus, which in turn leads to upregulation of *MS4A6A* expression and increased AD risk. rs636317-T is an hQTL for multiple enhancers in monocytes and a strong eQTL for *MS4A6A* in monocytes and macrophages, reinforcing the hypothesis that rs636317-T causes epigenetic dysregulation in the locus, which in turn may lead to increased expression of *MS4A6A*. Examination of promoter-capture Hi–C interactions in this region in monocytes and macrophages identified chromatin loops that connect the *MS4A6A* promoter to regulatory elements approximately 360 kilobases away (Fig. 4a (vi)). Importantly, examination of ChIA-PET interactions for CTCF and RAD21 (a component of the cohesin complex often colocalized with CTCF at anchor sites to form chromatin loops^47^) in GM12878 identified a chromatin loop that contains the *MS4A6A* promoter and connects two CTCF/RAD21 anchor sites, one of which is likely disrupted by rs636317-T (Fig. 4a (vii-ix)). This arrangement suggests that rs636317-T may alter chromatin architecture in such a way that the promoter of *MS4A6A* may lose its interaction with the regulatory elements mentioned above and instead fall under the influence of other regulatory elements that may boost *MS4A6A* expression in myeloid cells. Another established role of CTCF is the separation of regions of inner condensed chromatin and outer open chromatin domains, marking repressed and active regions, respectively^47^. Hence, we examined the density of epigenetic signals within and outside the CTCF/RAD21 loop boundaries in monocytes, macrophages and microglia (Fig. 4a (ii-iv)) and observed that chromatin activity within the loop is repressed. To gather additional experimental evidence in support of the epigenetic effects of this genetic polymorphism that we predicted based on computational analysis of experimental data obtained in a B-lymphoblastoid cell line or primary myeloid cells from peripheral blood, we investigated whether the C to T variation at rs636317 results in differential chromatin accessibility at this site in human microglia. To accomplish this, we generated hiPSC-derived microglia (Fig. 4c) from 3 subjects, performed ATAC-Seq and quantified the number of reads that correspond to the protective and risk-increasing alleles. We observed a significant difference in the number of normalized ATAC-Seq reads overlapping rs636317 with the protective allele (C) compared to the risk-increasing allele (T) (*P*-value = 0.007, paired one-sided t-test) (Fig. 4d). To test whether rs636317-T also leads to an increase in *MS4A6A* expression, we performed RNA sequencing in 4 hiPSC-derived microglia samples. We identified a single synonymous exonic variant in the *MS4A6A* gene (rs12453-C) that is in high LD with the risk variant in the CTCF binding site (rs636317-T, R^2^ = 0.92, ADGC reference panel was used to compute LD). Allele specific expression analysis revealed a difference in the number of normalized reads aligned to the T allele versus the C allele that was trending to significance (*P*-value = 0.088, one-sided paired t-test) (Methods). The direction, however, was the opposite of what is predicted by our analyses using primary myeloid cells from peripheral blood. This phenomenon has also been observed in a recent study showing that in another AD risk locus (*PTK2B*) the direction of the eQTL effect is flipped in hiPSC-derived macrophages as compared to primary blood monocytes and brain microglia^42^. These observations suggest that hiPSC-derived microglia might not be the best model for in-depth studies of the effects of genetic variation on gene expression and chromatin architecture at the *MS4A* and other AD risk loci.

Since a recent single-cell ATAC-seq study in the brain revealed that rs636317 resides in a microglia specific ATAC-seq peak^49^, we utilized brain ATAC-seq data from CommonMind^50^ to test if the ATAC-seq imbalance that we observed in hiPSC-derived microglia can be replicated in primary brain microglia. Indeed, we saw a significant imbalance in normalized ATAC-seq reads consistent with our computational and experimental data (*P*-value = 0.006, one-sided paired t-test) (Fig. 4e). Since expression of *MS4A6A* is also highly specific to microglia in the brain^15^, we performed allele-specific gene expression analysis using brain RNA-seq data from CommonMind (Methods). We observed a significant allelic imbalance (*P*-value=0.002, one-sided paired t-test) that is consistent with the direction of effect that we predicted using primary myeloid cells from peripheral blood (Fig. 4f-g). We were able to replicate this effect in the Mount Sinai Brainbank (MSBB)^51^ RNA-seq dataset, where we also observed a significant allelic imbalance (*P*-value = 3.0e-5, one-sided paired t-test). These results are consistent with a model in which the presence of the rs636317-T AD risk-increasing allele leads to disruption of CTCF binding, decreased chromatin accessibility at this site, altered chromatin looping and activity in the locus, and increased expression of *MS4A6A* in microglia. Further investigation of the mechanistic details of this model will require better human microglia culture systems or the use of acutely isolated primary microglia from the brain of larger numbers of human subjects or human-mouse chimeras^46,52,53^.

### Discussion

In this study we report an integration of AD GWAS with epigenomic and transcriptomic datasets from myeloid cells to nominate candidate causal variants, regulatory elements, genes and pathways and thus inform a mechanistic understanding of AD genetics and pathobiology for the formulation of novel therapeutic hypotheses (Supplementary Figure 7). Previous studies have shown that myeloid cells are the most disease-relevant cell type for AD^7,13^ and our own earlier study showed an enrichment of AD SNP heritability in myeloid-specific epigenomic annotations including the PU.1 cistrome^12^. Here we have extended these observations to demonstrate that AD risk alleles are specifically enriched in active enhancers of monocytes, monocyte-derived macrophages and microglia. Concordant with previous studies^15,20^, we show that PU.1, AP-1, C/EBP, CTCF, and RUNX binding motifs are overrepresented in open chromatin regions associated with active enhancers in all three myeloid cell types, while MEF2 transcription factor binding motifs are highly overrepresented in open chromatin regions associated with microglial active enhancers. To identify transcription factor binding sites burdened by AD risk variants, we stratified open chromatin regions that overlapped with myeloid active enhancers by the presence of cognate consensus motifs for the TFs mentioned above and quantified the enrichment of AD risk alleles in these subsets. A significant enrichment was observed in PU.1 binding motif-positive ATAC-Seq regions in all three myeloid cell types, while MAF binding motif-positive open chromatin regions were specifically enriched in macrophages and microglia. Furthermore, a significant enrichment of AD risk alleles was observed in SMAD, USF and SP binding motif-positive ATAC-Seq regions in microglia. These results suggest that AD risk variants are likely to modify disease susceptibility, at least in part, by modulating the binding of TFs to their cognate sequences in myeloid enhancers thus affecting their activity and in turn leading to target gene expression dysregulation. Although the global enrichment of AD risk alleles in active enhancers of myeloid cells narrows the search space for causal regulatory elements, identifying the target genes of these enhancers would directly point to candidate causal genes in AD risk loci.

**Fig. 5:**
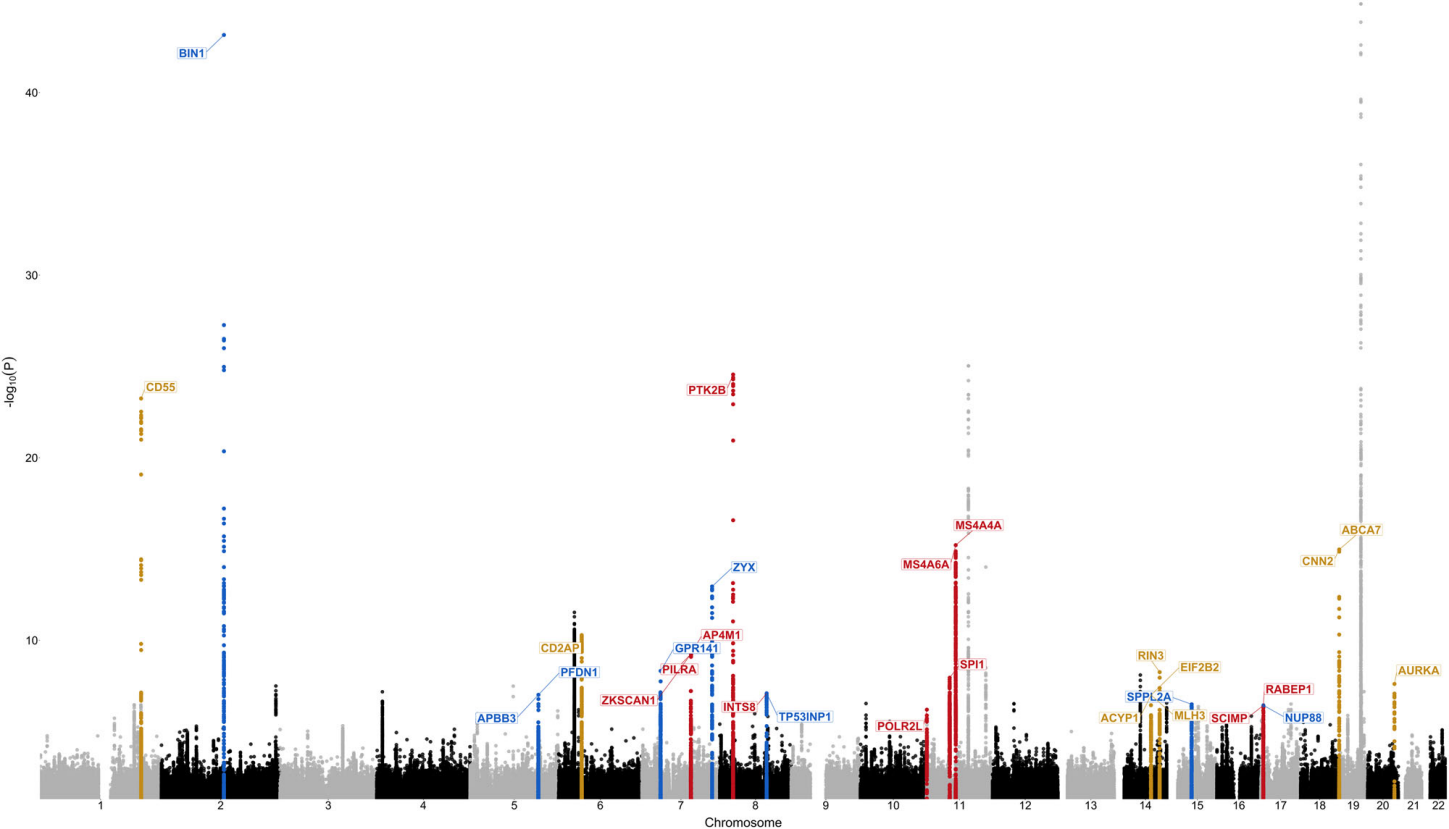
Candidate causal genes nominated through both Hi–C and SMR approaches in twenty loci. The Manhattan plot depicts the IGAP GWAS signal with putative AD risk genes assigned to each locus through both Hi–C and SMR approaches. Red indicates that increased expression of the gene is predicted to increase risk for AD. Blue indicates that decreased expression of the gene is predicted to increase risk for AD. Gold indicates that the directionality of gene expression that is associated with increased disease susceptibility cannot be robustly inferred. These genes were prioritized if they either a) interact with an AD risk enhancer that contains an eQTL for this gene or b) were implicated in enhancer activity to gene expression association, but did not have significant expression to disease risk associations (SMR). ZYX and PTK2B showed opposite directions of expression associated with disease risk in monocytes and macrophages. Strongest associations are reported (macrophages in ZYX locus and monocytes in PTK2B locus). The TREM2 locus is not shown since a well replicated rare loss-of-function mutations were found in TREM2^3^. The PICALM locus is not shown since the prioritized gene is not expressed in microglia.

In this study we used two complementary approaches to prioritize candidate causal target genes of myeloid active enhancers in AD risk loci. First, we mapped AD risk enhancers to their target genes in myeloid cells using chromatin interactions (Hi–C) and eQTL datasets from monocytes and macrophages. Using this approach, we identified previously nominated AD risk genes (*BIN1*^26^*, MS4A6A*^12^*, SPI1*^12^) as well as novel candidate causal genes including *AP4E1, APPB3, RIN3, TP53INP1,* and *ZYX* in sixteen loci. In a subset of AD risk loci we report shared active enhancers that interact with multiple target gene promoters to likely regulate their expression. This could reflect the presence of either multiple causal genes at these loci or a single causal gene and several risk neutral genes that show association by virtue of expression co-regulation. Additional evidence will be necessary to distinguish between these two possibilities and prioritize one or more genes at these loci. Second, we used SMR to test the causal relationships between activity at myeloid active chromatin regions with target gene expression regulation and AD risk modification. We sequentially studied the path linking active chromatin region activity with gene expression in myeloid cells using myeloid hQTLs as the exposure and myeloid eQTLs as the outcome, followed by myeloid eQTLs as the exposure and AD diagnosis as the outcome to identify regions that likely modulate AD risk by regulating the expression of one or more of their target genes in myeloid cells. Using this approach, we identified previously nominated AD risk genes *MS4A4A*^12^*, MS4A6A*^12^*, SPI1*^12^, as well as novel candidate causal genes *AP4E1, AP4M1, PILRA, RABEP1, SPPL2A, TP53INP1, ZKSCAN1*, and *ZYX* in twelve loci. Importantly, these two analytical approaches yielded largely overlapping results and led to the nomination of several candidate causal genes in twenty loci (Fig. 5). In all twenty loci we mapped candidate causal genes by identifying target genes of AD risk enhancers either through Hi–C interactions or chromatin activity to gene expression SMR associations. For those loci where the gene expression to disease risk association was significant, we were able to assign the directionality of AD risk gene expression that is associated with increased disease susceptibility (blue for lower expression and red for higher expression). The genes that did not show a significant expression to disease risk association but were prioritized through Hi–C interactions or chromatin activity to gene expression SMR associations, are shown in gold, since a causal association and its directionality cannot be robustly inferred. Moreover, in some of these loci both analytical approaches pointed to the same candidate causal genes (i.e. *BIN1*, *MS4A, SPI1, RABEP1, TP53INP1*, and *ZYX*). Remarkably, when a *BIN1* enhancer prioritized through our approaches was deleted in hiPSC-derived microglia, neurons and astrocytes, *BIN1* expression and protein level dramatically decreased only in microglia, underpinning cell type-specific regulatory potential of a rather ubiquitously expressed gene and pointing to the robustness of our findings^46^.

Notably, many of the candidate causal genes that we identified in myeloid cells are functionally related to the endolysosomal system. For example, *ZYX* encodes a zinc-binding phosphoprotein that localizes to early endosomes and phagosomes in IFN-γ-activated macrophages^54^ and drives their intracellular movement by assembling actin filament rocket tails^55^. *RIN3* (Ras And Rab Interactor 3) encodes a member of the RIN family of RAS and RAB effectors that interacts and localizes with BIN1 to early endosomes^56^. Like other RIN family members, RIN3 has guanine nucleotide exchange factory (GEF) activity for RAB5 GTPases^56^, which are required for early endosome and phagosome biogenesis and function. Interestingly, *RABEP1* (Rab-GTPase binding effector protein 1) also encodes a RAB5 effector protein that is required for early endosome membrane fusion and trafficking^57^. Two other novel candidate AD risk genes that we nominated in this study, *AP4E1* and *AP4M1*, encode two of the four subunits of the heterotetrameric adaptor protein complex 4 (AP-4), which is required for the sorting of transmembrane proteins like APP from the trans-Golgi network (TGN) to endosomes^58^. Interestingly, APBB3 has also been shown to bind to the intracellular domain of APP and is thought to play a role in the internalization of APP from the cell surface into endosomes where it is cleaved by membrane-embedded aspartyl proteases BACE1 and ɣ-secretase to generate the amyloid β peptide^59,60^. Another novel candidate AD risk gene that we nominate in this study, *SPPL2A*, encodes a transmembrane aspartyl protease that localizes to late endosomes and lysosomes and cleaves substrates involved in immunity and neurodegeneration^61–63^. Finally, *TP53INP1* regulates the stability and transcriptional activity of p53, and has been implicated in the phagocytic clearance of apoptotic cells (efferocytosis)^64,65^, a hallmark function of macrophages for the maintenance of tissue homeostasis and immune tolerance, and the resolution of inflammation. All of these genes are highly or selectively expressed in microglia in the brain^15^. Taken together, our findings implicate dysfunction of the endolysosomal system in myeloid cells (as opposed to neurons^66^) in the etiology of AD. Previous human genetic findings reinforce our conclusion. For example, a rare variant in the 3′ UTR of *RAB10*, a member of the RAB family of small GTPases that are critical regulators of membrane trafficking and vesicular transport, confers resilience to AD^67^. Furthermore, coding variants that increase risk for AD have been identified in *SORL1*^4,68^, a member of the vacuolar protein sorting 10 (VPS10)- domain-containing receptor family and the low density lipoprotein receptor (LDLR) family of APOE receptors that is expressed primarily in microglia in the brain^15^ and plays important roles in the endolysosomal system and APP processing^66^.

To fine-map the AD risk enhancers identified in this study and thus nominate candidate causal variants, we conducted Bayesian fine-mapping in the three loci that were significantly associated with AD risk in the ADGC GWAS (*BIN1, MS4A*, and *ZYX*), followed by functional in silico screening of the candidate causal variants for disruption/creation of TF binding motifs. We also fine-mapped the loci that did not reach significance in the ADGC GWAS (but were significant or suggestive in the IGAP GWAS) and identified candidate causal variants in the *GPR141, RABEP1, SPI1*, and *SPPL2A* loci. Taken together, we have identified putative functional variants that tag the majority of AD GWAS signals at these loci, and likely affect disease risk by altering the DNA binding motifs of transcription factors that modulate the activity of enhancers which in turn regulate the expression of causal genes to ultimately steer myeloid cells like microglia toward neurotoxic and/or away from neuroprotective phenotypes. Finally, we experimentally validated one of these candidate functional variants in the *MS4A* locus by showing allelic imbalance in open chromatin in hiPSC-derived microglia as well as in open chromatin and *MS4A6A* mRNA levels in the brain. The epigenetic effects of this variant are likely mediated by the disruption of CTCF binding at one of two anchor sites of a repressive chromatin loop leading to increased *MS4A6A* expression and AD risk, although investigation of the mechanistic details of this model will require further experimentation.

Our analyses demonstrate that active enhancers in monocytes, macrophages and microglia are enriched significantly and to a similar extent. These results provide evidence that AD risk alleles burden regulatory sequences similarly across all three myeloid cell types and that the basal state is, at least in part, relevant to the study of regulatory variants that affect AD risk. Recent findings that TREM2 loss of function similarly impacts the response of both central nervous system (CNS) and peripheral macrophages to lipid overload^69–71^ and that the activation state of human macrophages does not have a major impact on AD heritability enrichment^72^ could indicate that Alzheimer’s disease-associated variants might regulate core functions of the macrophage lineage (e.g., the phagocytic clearance of apoptotic cells and other lipid-rich cellular debris). These results highlight the need to generate additional large-scale human microglial/myeloid epigenomic and transcriptomic datasets (e.g., in the context of immune and metabolic stress) which will enable identification of the most disease-relevant myeloid cell states and enable replication and extension of our findings.

The integrative genomic approaches presented here offer a framework to identify regulatory elements, genes and variants that are likely causal for AD. A potential limitation of our study is that integration of epigenomic and transcriptomic datasets from different studies using varying protocols for the isolation and preparation of monocytes and macrophages, might lead to false positive and negative results in some of our analyses. This highlights the need for paired epigenomic and transcriptomic datasets in myeloid cells to further validate and expand our findings. Further experimental validation of the variants and enhancers nominated in this manuscript will be needed to dissect the molecular mechanism of action as well as downstream effects in myeloid cells. Using our prediction as a guiding tool, CRISPR experiments can be performed to test the effects of a single variant or regulatory elements in isogenic lines on TF binding, gene expression and downstream myeloid cell biology, e.g phagocytosis of lipid-rich debris. Additionally, recent studies have demonstrated that iPSC-derived microglia can be transplanted into the mouse brain while recapitulating expression profiles of human primary microglia^52^. These advances can be utilized to transplant iPSC-derived microglia lines with CRISPR induced alterations to study the effects of non-coding AD risk variants and regulatory elements in vivo.

In summary, this study reveals a link between chromatin activity, gene expression and AD risk in myeloid cells, proposes the molecular mechanism of action of candidate functional variants in several AD risk loci, identifies specific AD risk enhancers that are burdened by these variants and regulate target gene expression, which in turn most likely modulates disease susceptibility by altering the biology of myeloid cells. We highlight the coalescence of candidate causal genes in the endolysosomal system of myeloid cells and underscore its importance in the etiology of AD.

## Methods

### Processing of ChIP-Seq and ATAC-Seq data and peak calling

Relevant ChIP-Seq and ATAC-seq studies were found through Gene Expression Omnibus (GEO)^15,36,38,73^. We selected studies that contained H3K4me1 (monocytes and macrophages), H3K4me2 (monocytes and microglia) and/or H3K4me3 (macrophages) as well as H3K27ac (all cell types) and ATAC-seq (all cell types) data for human monocytes, macrophages and microglia for our analyses. To generate the epigenomic annotations FASTQ files were obtained from Sequence Read Archive (SRA). Technical replicates were merged and Bowtie2^74^ was used for alignment for both single and paired-end files. FASTQC was used for quality control of the files. Resulting SAM files were filtered by MAPQ score and duplicates were removed using samtools^75^. MACS2^76^ was used to call peaks for ATAC-seq and ChIP-seq files. ATAC-Seq peaks were called using the following command: “callpeak -t file.sam -f SAM --nomodel --shift -37 --extsize 73 -g hs -q 0.01 -n filename --outdir output_dir/”. PU.1 ChIP-Seq peaks were called using the following command: callpeak -t case.sam -c input.sam -f SAM -g hs -q 0.01 -n filename --outdir output_dir/”. Histone modifications ChIP-Seq peaks were called using the following command: “callpeak -t case.sam -c input.sam -f SAM --broad --broad-cutoff 0.01 -g hs -q 0.01 -n filename --outdir output_dir/”.

### Stratification into promoter and enhancer regions and overlap with GWAS and Hi–C data

To identify optimal distance from TSS we used ChromHMM model of CD14 + monocytes from Roadmap Epigenomics project (see URLs) to visualize the distribution of active promoters around the TSS. We observed a bimodal distribution around the TSS and found that −500 base pairs to 1000 base pairs window captures more than 60% of active promoters. Based on previous studies that have demonstrated a bimodal distribution of promoter epigenomic marks around the TSS^77,78^, we established that the boundary of −500, 1000 bp would appropriately mark active promoters, while also not misclassifying H3K4me1 positive regions (enhancers) that are in close proximity to the TSS. To annotate the peaks with distance from TSS we used HOMER. We then split the H3K4me1/2/3 peaks into distal and proximal. We then used bedmap to filter H3K4me1/2/3 peaks by the presence of H3K27ac peak such that proximal H3K4me2/3 peaks with H3K27ac were classified as active promoters, distal H3K4me1/2 peaks with H3K27ac were classified as active enhancers, proximal H3K4me2/3 peaks without H3K27ac were classified as primed promoters and distal H3K4me1/2 peaks without H3K27ac were classified as primed enhancers. AD risk enhancers were identified by overlapping active enhancers (including a 500-bp flanking region on each side) with AD risk alleles (*P* ≤ 1 × 10^−6^). To identify likely targets of AD risk enhancers, enhancers (including a 3000-bp flanking region on each side) were overlapped with Hi–C target regions that showed evidence of regulatory effect (eQTL FDR 5%).

### Partitioned SNP-heritability analysis

We used LD Score regression to estimate AD SNP heritability partitioned by epigenomic annotations using GWAS summary statistics (excluding the APOE (chr19:45000000– 45800000) and MHC/HLA (chr6:28477797–33448354) regions) in myeloid cells as described in the companion website (see URLs), while controlling for the 53 functional annotation categories of the full baseline model. GWAS summary statistics for AD^17^ and Schizophrenia^18^ (SCZ) were downloaded from the IGAP Consortium and Psychiatric Genomics Consortium websites respectively (see URLs). All epigenomic annotations were downloaded from SRA and processed as described in “Processing of ChIP-Seq and ATAC-Seq data and peak calling”. Negative log10 *p*-values of enrichment were reported, the *p*-values for annotations that had negative enrichments were not displayed on the figures.

### De novo motif discovery

We used HOMER to perform de novo motif discovery in ATAC-Seq regions that reside in active enhancers in monocytes, macrophages and microglia. The following command was used to identify enriched motif sequences in these regions: findMotifsGenome.pl Peaks.bed hg19. -size given. To identify regions that contained our motifs of interest, we used the following commands: findMotifsGenome.pl Peaks.bed hg19. -find motif.motif -size given and annotatePeaks.pl Peaks.bed hg19 -m motif.motif -size given.

### Colocalization analysis

We used coloc (coloc.abf function) to perform colocalization analyses between IGAP GWAS and hQTLs with default parameters^29^. We used coloc in the following manner: coloc.abf(dataset1, dataset2, p1 = 1e-04, p2 = 1e-04, p12 = 1e-05). We used a filter of PP.H3.abf + PP.H4.abf ≥ 0.8 to select chromatin regions with evidence of independent or colocalized AD GWAS and hQTL signals.

### Causal association analysis

We used SMR to test for causal associations between IGAP GWAS and QTL datasets^14^. We converted the summary statistics for monocyte H3K4me1 hQTLs obtained from BLUEPRINT epigenome project website (see URLs) and monocyte eQTLs from the Cardiogenics and Fairfax studies into BESD format (epi/esi/besd) as described in the SMR manual (see URLs). Allele frequencies and LD were estimated from the ADGC GWAS cohort individual-level genotype data using plink^79^. To conduct standard SMR analysis, we ran the following command: “smr --bfile reference_file --beqtl-summary Exposure_besd_file_prefix --beqtl-summary Outcome_besd_file_prefix --out output_prefix”. The results were filtered for FDR of 5% calculated using the p.adjust function in R. To conduct SNP-targeted SMR analysis, we ran the following command: “smr --bfile reference_file --gwas-summary gwas_summary_file --beqtl-summary eQTL_besd_file_prefix --target-snp rs12345 --out output_prefix”.

### Conditional and haplotype analyses

We used GCTA-COJO^41^ to conduct conditional analyses using IGAP GWAS summary statistics data and ADGC GWAS cohort individual-level genotype data as a reference panel. To conduct the conditional analysis we ran the following command: “gcta64 --bfile reference_file --maf 0.05 --cojo-file GWAS_summary_statistics --cojo-cond list_of_snps --out output_prefix”. To construct haplotype blocks and examine SNP clustering, we used Big-LD^40^ which is provided as an R package. We prepared the genotype file, which contained genotypes of individuals for each SNP, and the SNP information file that contained chromosome, position, reference and alternative allele information for each SNP. We then used the CLQ algorithm provided within the Big-LD package for SNP clustering and BigLD for haplotype block construction. We used the following commands: CLQD(geno = genotype_data, SNPinfo = locus_snp_information, hrstType = “fast”,CLQcut = 0.5) and Big_LD(genotype_data, SNPinfo = locus_snp_information, chrN = chromosome_number, startbp = start_basepair, endbp = end_basepair, appendRare = TRUE).

### Prioritization of candidate causal variants

For each locus we constructed LD blocks using Big-LD package^40^. We then selected variants that reside in active enhancers in monocytes, macrophages and/or microglia (with the exception of the *SPPL2A* locus, since these variants likely regulate a distal enhancer as reported in Fig. 3c). We also conducted a motif disruption/creation analysis on these variants and selected the variants that are predicted to strongly disrupt or create binding sites of transcription factors that are expressed in myeloid cells (TPM ≥ 1)^15^. We then screened the remaining variants for eQTLs in monocytes and macrophages from the Cardiogenics and Fairfax studies. We also used PAINTOR to conduct Bayesian fine-mapping in MS4A, ZYX and BIN1 loci. PAINTOR is a Bayesian fine-mapping method that leverages functional annotations through an Empirical Bayes prior^32^. The input files for PAINTOR (v3.1) were prepared as described on the PAINTOR website and ADGC GWAS summary statistics along with individual-level genotype data were used for fine-mapping (see URLs). The reprocessed epigenomic annotations were used to quantify enrichment at each locus. To quantify the annotation enrichments the following command was used: “python AnnotateLocus.py --input list_of_annotation_directories --locus locus_prefix --out output_prefix --chr chr --pos pos”. To classify the annotations as enriched or not, we computed the relative probability for a SNP to be causal given that it resides in the annotation as described in the companion website (URLs). We deemed the annotation to be significant if the relative probability of a SNP to be causal given that it is in the annotation was greater than 1. To quantify the posterior probabilities for variants to be causal, we used the following command: PAINTOR -input input.file -Zhead Zscore -LDname ld -enumerate max_number_of_causal_variants -annotations annotation_name -in in_dir -out out_dir. Once candidate causal variants were selected through both approaches, we conducted conditional analyses to make sure that they do indeed tag the majority of the GWAS signal in the locus.

### TF binding motif disruption/creation analysis

We used motifbreakR to predict the impact of AD risk variants on transcription factor binding^39^. We used HOCOMOCO to screen for TFBMs and a *P*-value significance threshold of 5 × 10^−5^. We used the following command to do so: motifbreakR(snpList = variant_list, pwmList = hocomoco, filterp = TRUE, threshold = 5e-5).

### Generation of hiPSC microglia for ATAC-Seq and RNA-seq analysis

hiPSC-derived microglia were generated from patient lines following the protocol as described^80^. For the ATAC-Seq analysis, hiPSC-derived microglia (50 K cells) from each patient line were collected and processed as described^81^. Samples were either processed at New York Genome Center or at UCI’s Genomics High-Throughput Facility and sequenced as 50 bp paired-end reads on a HiSeq 2500 and 100 bp paired-end reads on a HiSeq 4000, respectively. The consent for reprogramming patient somatic cells to hiPSC was carried out on protocol 2013-9561 (UCI), laboratory protocol 2017-1061 (UCI) and protocol ESCRO 19-04 (Mount Sinai). Microglia RNA was isolated using a standard RNA isolation kit (Qiagen) and RNA quality (RIN) assessed (Bioanalyzer 2100). PolyA-mRNA (200 ng) with a RIN score ≥ 9.5 was used to assemble libraries in which ERCC spike-ins (Ambion) were included for downstream normalization. RNA-seq libraries were quantified and normalized using a Library Quantification Kit (Kapa Biosystems) prior to sequencing (Illumina) by the UCI Genomics High Throughput Facility as 100 bp paired-end reads.

Human iPSC cell lines were either generated by the University of California, Irvine Alzheimer’s Disease Research Center (UCI ADRC) Induced Pluripotent Stem Cell Core or by the Icahn School of Medicine at Mount Sinai Induced Pluripotent Stem Cell Core. The iPSC lines generated by University of California, Irvine and the Icahn School of Medicine at Mount Sinai were derived from subject fibroblasts from either the University of California, Irvine or Washington University in St. Louis, respectively, with approved Institutional Review Boards (IRB) and human Stem Cell Research Oversight (hSCRO) committee protocols. Informed consent was received by each of the participants who donated fibroblasts.

### Allele specific expression and open chromatin analysis

In CommonMind and Mount Sinai Biobank (MSBB) datasets we selected the RNA-seq samples that contained at least 10 reads aligned to the SNP of interest. For CommonMind ATAC-seq samples, we required at least 5 reads aligned to the SNP of interest. To perform allele specific expression/open chromatin analyses, we have quantified the number of reads overlapping the variant of interest using mpileup command in samtools^75^. The CommonMind and MSBB reads were normalized to the number of reads on chromosome 11 and were used to assess the significance of the allelic imbalance using a paired t-test.

### Immunocytochemistry

Cells were fixed with 4% paraformaldehyde in PBS at 4 °C for 10 min. Cells were permeabilized with 1.0% Triton in PBS at room temperature for 15 min and blocked in 5% donkey serum with 0.1% Triton in PBS at room temperature for 30 min. Primary antibodies were used at 10 µg/mL anti-TREM2 (R&D, AF1828), 1:1,000 anti-P2RY12 (Sigma, HPA014518), 1:100 anti-PU.1 (Cell Signaling, 2266) and anti-CX3CR1(Bio-Rad, AHP1589). Secondary antibodies were used at 1:300 Alexa donkey 488 and 568 anti-rabbit, mouse, or chicken (Life Technologies). DAPI (4′,6-diamidino-2-phenylindole, 0.5 μg/mL) was used to visualize nuclei. Images were acquired using a Leica Fluorescence Microscope at 40× magnification.

### Reporting summary

Further information on research design is available in the Nature Research Reporting Summary linked to this article.

## Supplementary information

Supplementary Information

Description of Additional Supplementary Files

Supplementary Data 1

Supplementary Data 2

Supplementary Data 3

Supplementary Data 4

Supplementary Data 5

Supplementary Data 6

Supplementary Data 7

Supplementary Data 8

Reporting Summary

## Data Availability

The following studies obtained from GEO were used for the analyses presented in this paper: GSE29611 (monocyte CTCF, H3K27ac and H3K4me1/2/3), GSE85245 (macrophage H3K27ac and H3K4me1/3), GSE100380 (monocyte and macrophage ATAC-seq), GSE66594 (macrophage H3K4me1, H3K27ac and PU.1) and GSE98365 (macrophage ATAC-seq). Data generated in this study are available through accession number GSE164315. DbGAP accession study number for the human microglia dataset is phs001373.v2.p2. The genotype and phenotype data from ADGC are available under phs000372.v1.p1 dbGAP study accession number. The Cardiogenics dataset can be requested on EGA using accession number EGAS00001000411. DbGAP accession study number for the STARNET eQTL dataset is phs001203.v1.p1. Summary statistics for Fairfax eQTL data can be obtained from ArrayExpress using accession number E-MTAB-2232. All data supporting the findings of this study are provided within the paper and its supplementary information. All other relevant data are available from the authors upon reasonable request.

## Code availability

We provide commands for the tools that were used for the analyses presented in this manuscript in the Methods section. Although we have used the software cited in this manuscript with default parameters or minor changes, code for these analyses is available upon request. Roadmap Epigenomics Project, http://www.roadmapepigenomics.org/ - The dataset was used to examine the distribution of active promoters around the TSS in the ChromHMM model of CD14 + monocytes to identify an appropriate window to stratify enhancers and promoters. LD Score Regression, https://github.com/bulik/ldsc; - LDSC was used to quantify the enrichment of AD heritability in myeloid epigenomic annotations. International Genomics of Alzheimer’s Project (IGAP), http://web.pasteur-lille.fr/en/recherche/u744/igap/igap_download.php; - The summary statistics for AD GWAS were obtained from IGAP. Psychiatric Genomics Consortium, https://www.med.unc.edu/pgc/; - SCZ GWAS summary statistics were obtained from PGC. Blueprint Consortium, http://www.blueprint-epigenome.eu/; - Monocyte hQTLs were obtained from the Blueprint Consortium. SMR, https://cnsgenomics.com/software/smr/; - SMR was used to test for candidate causal associations between chromatin activity, gene expression and disease risk. COJO, https://cnsgenomics.com/software/gcta/#COJO; - COJO was used to conduct conditional analyses. PAINTOR, https://github.com/gkichaev/PAINTOR_V3.0; - PAINTOR was used to conduct Bayesian fine-mapping analyses. ADGC, https://brightspotcdn.byu.edu/bf/be/75f2076b4241a30840eadda2c66c/adgc-combined-1000g-09192014.pdf; - ADGC genotype data were used for fine-mapping and as an LD reference panel. HOMER, http://homer.ucsd.edu/homer/; - HOMER was used for de novo motif enrichment analyses.
