# Supplementary material for: Integration of Alzheimer’s disease genetics and myeloid genomics identifies disease risk regulatory elements and genes
Source: Nat Commun. 2021 Mar 12;12:1610. doi: 10.1038/s41467-021-21823-y (PMC7955030; doi:10.1038/s41467-021-21823-y)
Supplement: Supplementary file 1 — Supplementary Information [file 41467_2021_21823_MOESM1_ESM.pdf]

Supplementary Figure 1 - Nomination of candidate causal genes in the ZYX locus using epigenomics, Hi-C, eQTL and GWAS data.

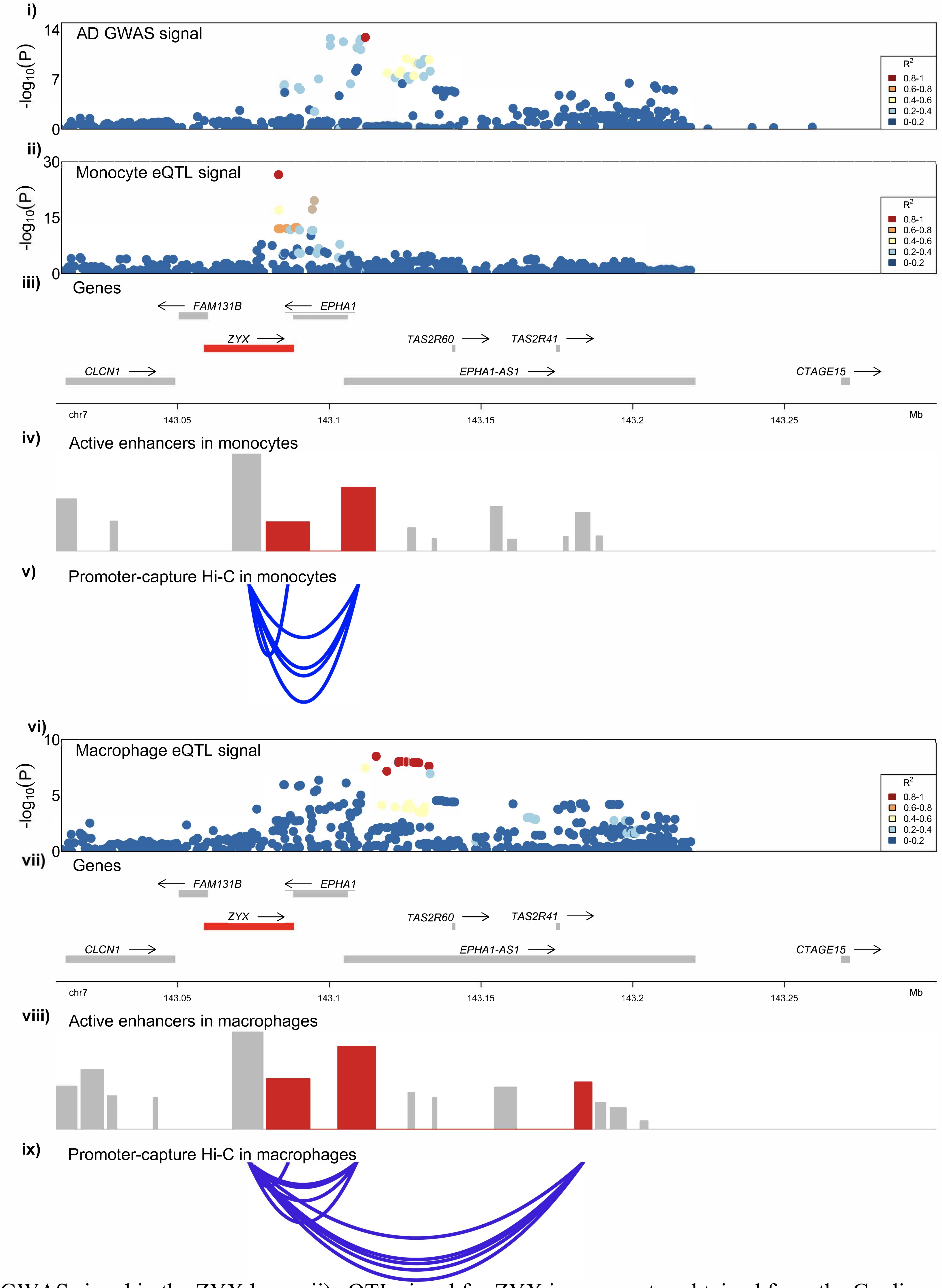

Supplementary Figure 1. i) AD GWAS signal in the ZYX locus. ii) eQTL signal for ZYX in monocytes obtained from the Cardiogenics study. iii) Genes that reside in the locus are plotted. Likely target genes of AD risk enhancers are highlighted in red. iv) Active enhancers in monocytes are plotted. AD risk enhancers that interact with the ZYX promoter in monocytes are highlighted in red. The height of the bar is proportional to the strength of the epigenomic signal. v) Promoter-capture Hi-C interactions between the ZYX promoter and the highlighted AD risk enhancers in monocytes. The depth of the arc is proportional to the strength of the interaction. vi) eQTL signal for ZYX in macrophages obtained from the Cardiogenics study. vii) Genes that reside in the locus are plotted. Likely target genes of AD risk enhancers are highlighted in red. viii) Active enhancers in macrophages are plotted. AD risk enhancers that interact with the ZYX promoter in macrophages are highlighted in red. The height of the bar is proportional to the strength of the epigenomic signal. ix) Promoter-capture Hi-C interactions between the ZYX promoter and AD risk enhancers in macrophages. The depth of the arc is proportional to the strength of the interaction. Hi-C interactions are anchored at the AD risk enhancers highlighted.

Supplementary Figure 2 - Nomination of candidate causal genes in the TP53INP1 locus using epigenomics, Hi-C, eQTL and GWAS data.

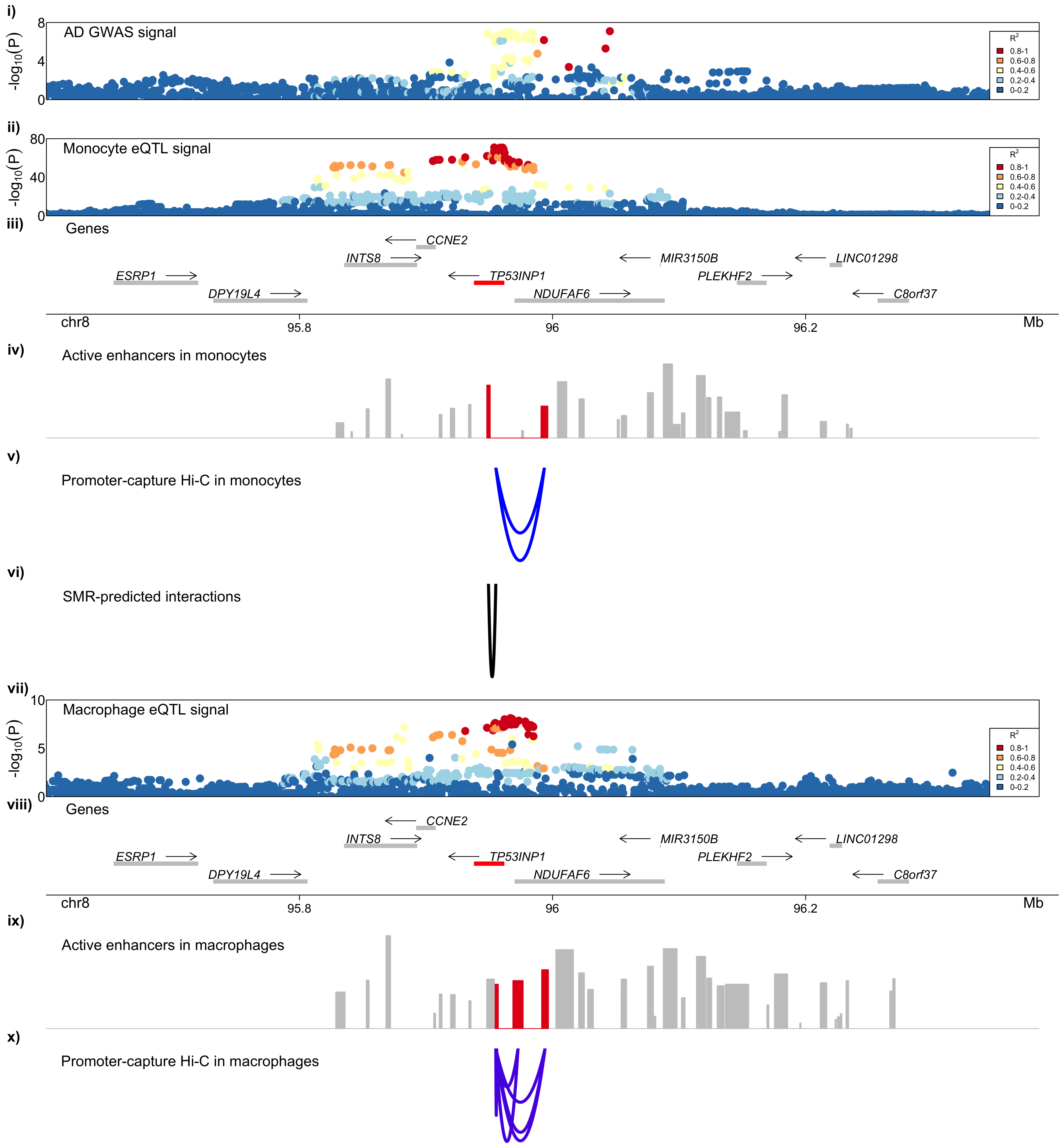

Supplementary Figure 2. i) AD GWAS association signal in the TP53INP1 locus. ii) eQTL signal for TP53INP1 in monocytes obtained from the Cardiogenics study. iii) Genes that reside in the locus are plotted. Likely target genes of AD risk enhancers shown are highlighted in red. The arrow indicates the direction of transcription, while the bar indicates the gene body. iv) Active enhancers in monocytes are plotted. AD risk enhancers that interact with the TP53INP1 promoter or prioritized through SMR are highlighted in red. The height of the bar is proportional to the strength of the epigenomic signal. v) Promoter-capture Hi-C interactions between the TP53INP1 promoter and AD risk enhancers in monocytes. The depth of the arc is proportional to the strength of the interaction. vi) AD risk enhancer-target gene interactions predicted by SMR analysis of causal associations between chromatin activity and TP53INP1 expression in monocytes. The depth of the arc is proportional to the strength of the association. vii) eQTL signal for TP53INP1 in macrophages obtained from the Cardiogenics study. viii) Genes that reside in the locus are plotted. Likely target genes of AD risk enhancers shown are highlighted in red. The arrow indicates the direction of transcription, while the bar indicates TP53INP1 gene body. ix) Active enhancers in macrophages are plotted. AD risk enhancers that interact with the TP53INP1 promoter are highlighted in red. The height of the bar is proportional to the strength of the epigenomic signal. x) Promoter-capture Hi-C interactions between the TP53INP1 promoter and AD risk enhancers in macrophages. The depth of the arc is proportional to the strength of the interaction. Hi-C and SMR-predicted interactions are anchored at the AD risk enhancers highlighted.

Supplementary Figure 3 - Conditional analysis in the PILRA locus using the coding candidate causal variant in the locus .

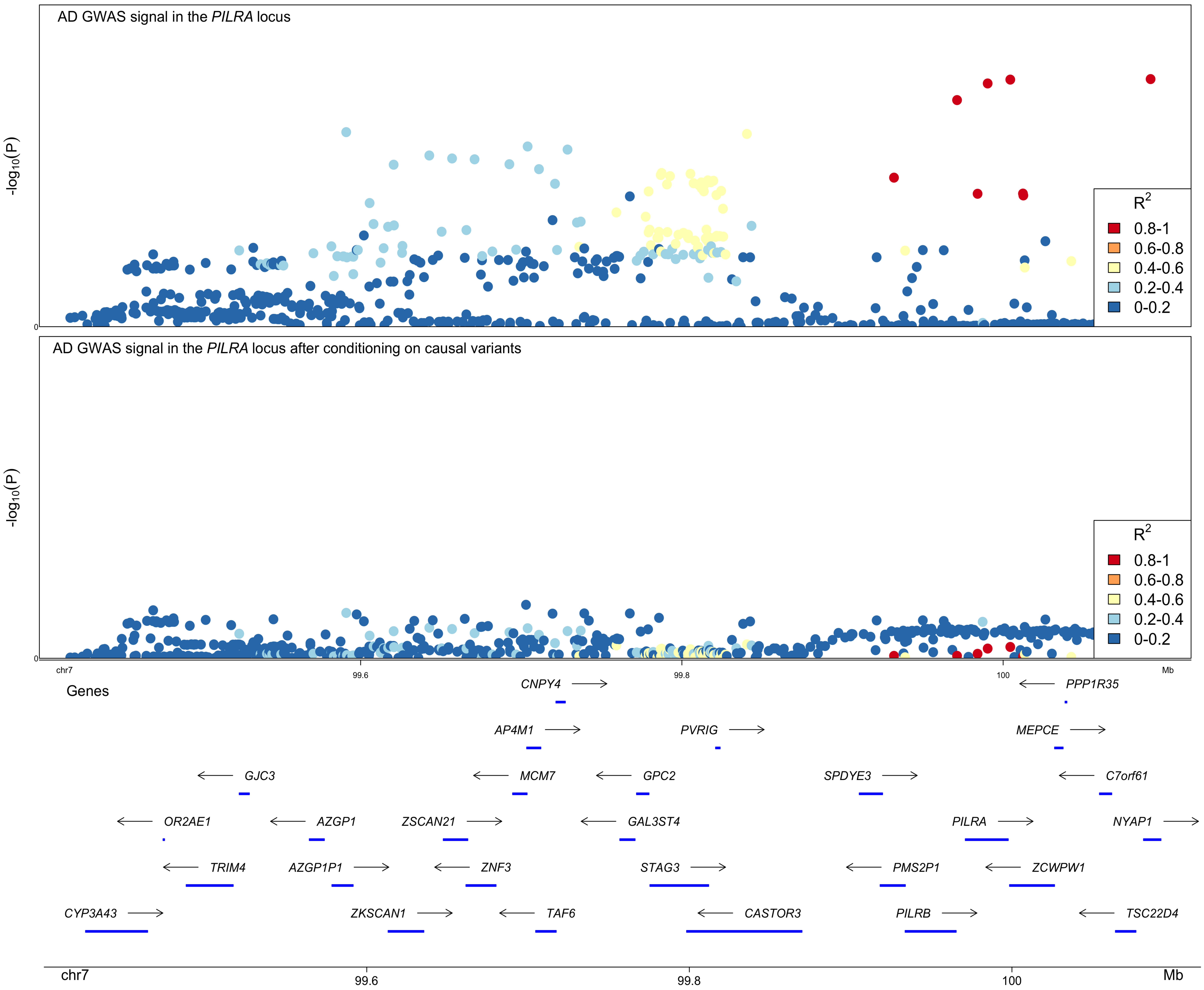

Supplementary Figure 3. Conditional analysis plots of the missense variant (rs1859788-G) in the PILRA locus.

Supplementary Figure 4 - Enrichment of epigenomic annotations using PAINTOR in MS4A, BIN1 and ZYX loci

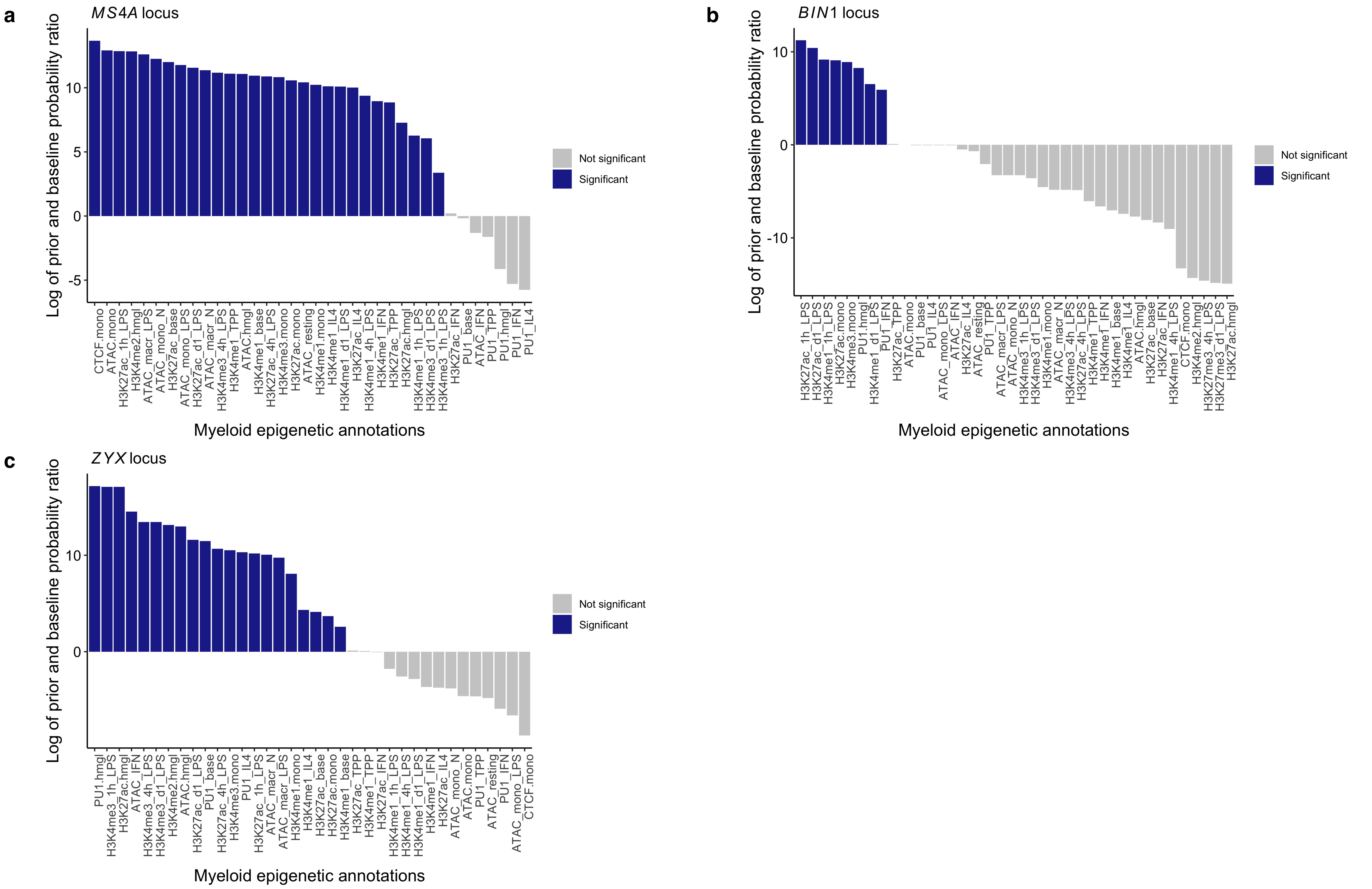

Supplementary Figure 4. Log10 of prior and baseline probabilities ratio obtained from PAINTOR fine-mapping analysis of the a) MS4A b) BIN1 and c) ZYX loci plotted for each myeloid epigenomic annotation tested. Annotations that are significantly enriched in the locus are colored in blue, while non-significant annotations are colored in grey.

Supplementary Figure 5 - Conditional analyses using candidate causal variants nominated for each respective locus

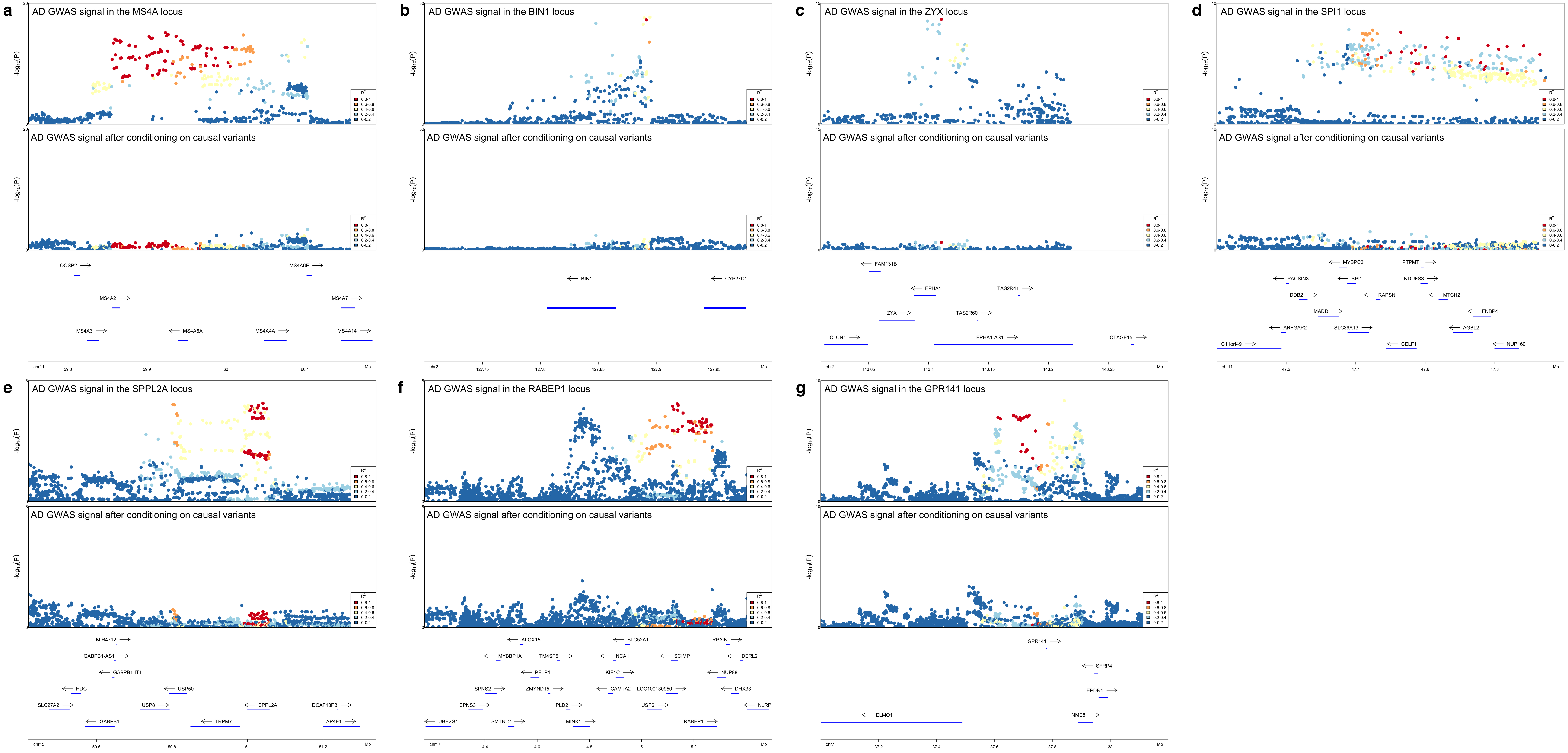

Supplementary Figure 5. Conditional analysis plots of the candidate causal variants listed in Supplementary Table 8 in the a) MS4A , b) BIN1 , c) ZYX , d) SPI1 , e) SPPL2A , f) RABEP1 and g) GPR141 loci.

Supplementary Figure 6 - Allelic imbalance in human brain for candidate causal variants in the BIN1 locus

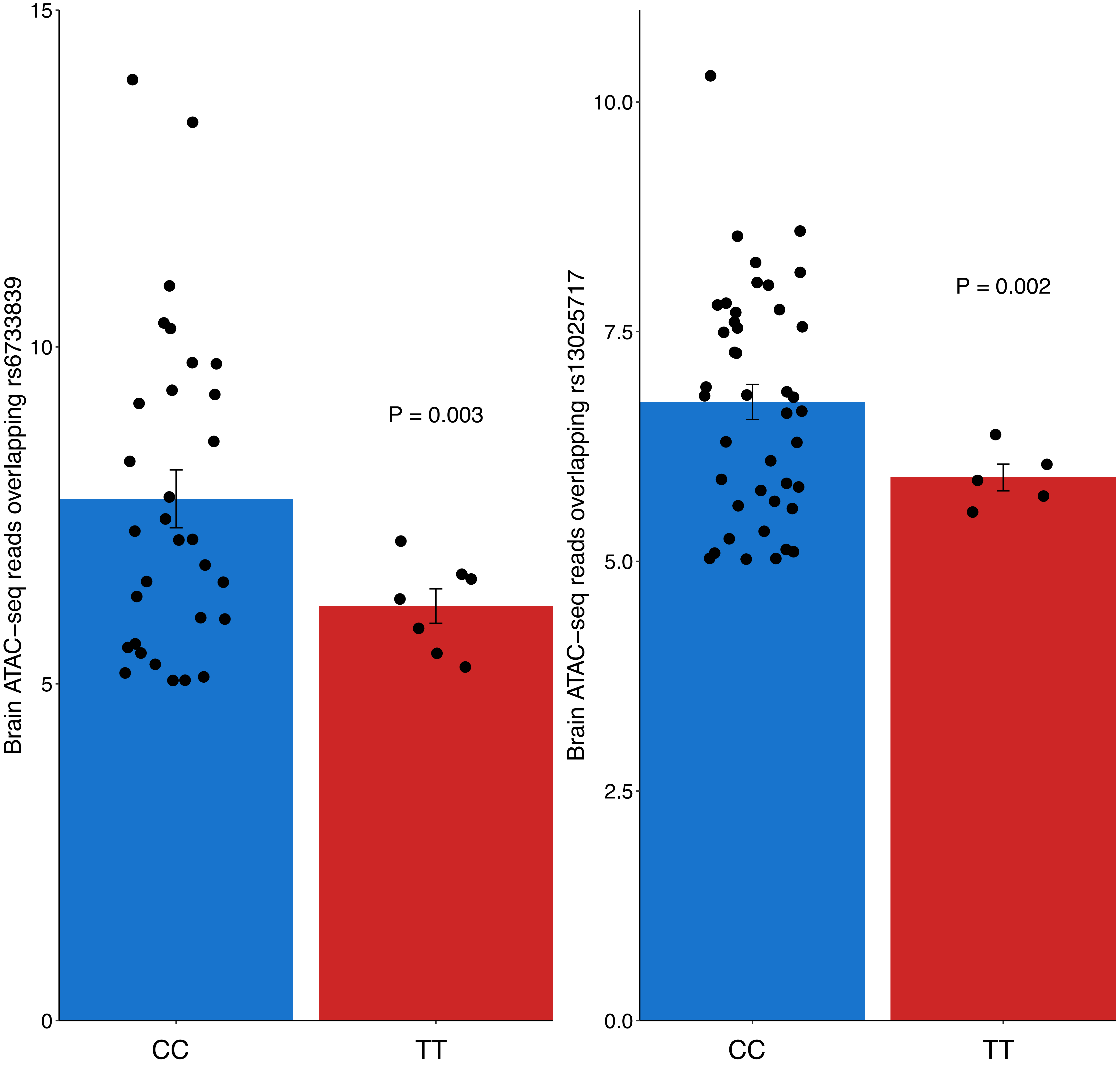

Supplementary Figure 6. a) ATAC-seq reads overlapping rs6733839 site in the brain in homozygous individuals. n = 7 homozygotes for T allele, n = 31 homozygotes for C allele. b) ATAC-seq reads overlapping rs13025717 site in the brain in homozygous individuals. n = 5 homozygotes for T allele, n = 42 homozygotes for C allele.

The bar heights represent mean ATAC-seq read counts for each allele, the dots represent each individual and error bars represent standard errors. A two-sided t-test was used to obtain p-values.

Supplementary Figure 7

Summary figure for the integrative genomic approaches we have utilized to discover candidate causal genes.

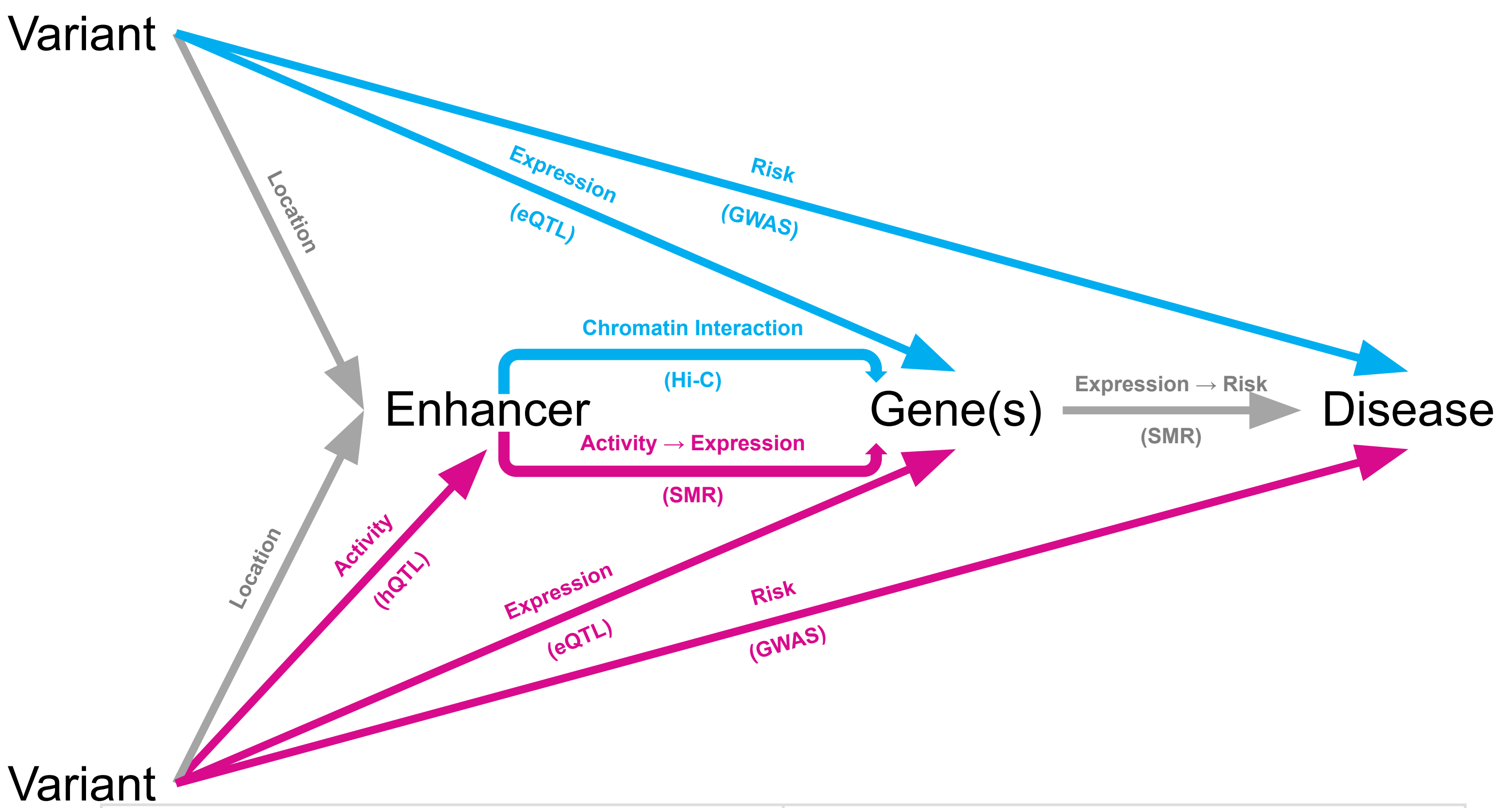

Monocytes	Macrophages
Fairfax and Cardiogenics eQTL<br>Javierre et al promoter-capture Hi-C<br>H3K4me1/2 and H3K27ac epigenetic marks	Cardiogenics and STARNET eQTL<br>Javierre et al promoter-capture Hi-C<br>H3K4me1 and H3K27ac epigenetic marks
Fairfax and Cardiogenics eQTL<br>Blueprint hQTL	-
