# Supplementary material for: Integration of Alzheimer’s disease genetics and myeloid genomics identifies disease risk regulatory elements and genes
Source: Nat Commun. 2021 Mar 12;12:1610. doi: 10.1038/s41467-021-21823-y (PMC7955030; doi:10.1038/s41467-021-21823-y)
Supplement: Supplementary file 2 — Description of Additional Supplementary Files [file 41467_2021_21823_MOESM2_ESM.pdf]

Description of Additional Supplementary Files

Title: Supplementary Data 1

Description: De novo motif analysis of monocyte, macrophage and microglial enhancers.

Title: Supplementary Data 2

Description: Colocalization results between monocyte hQTLs and AD GWAS.

Title: Supplementary Data 3

Description: SMR results for associations between monocyte hQTLs and eQTLs (Cardiogenics).

Title: Supplementary Data 4

Description: SMR results for associations between eQTLs (Cardiogenics) and AD GWAS.

Title: Supplementary Data 5

Description: SMR results for associations between monocyte hQTLs and eQTLs (Fairfax).

Title: Supplementary Data 6

Description: SMR results for associations between eQTLs (Fairfax) and AD GWAS.

Title: Supplementary Data 7

Description: Candidate causal variants nominated in AD risk loci through fine-mapping.

Title: Supplementary Data 8

Description: SNP-targeted SMR for candidate causal variants in monocytes and macrophages.
