# Supplementary material for: Integration of Alzheimer’s disease genetics and myeloid genomics identifies disease risk regulatory elements and genes
Source: Nat Commun. 2021 Mar 12;12:1610. doi: 10.1038/s41467-021-21823-y (PMC7955030; doi:10.1038/s41467-021-21823-y)
Supplement: Supplementary file 11 — Reporting Summary [file 41467_2021_21823_MOESM11_ESM.pdf]

nature research

Corresponding author(s): Alison Goate, Edoardo Marcora

Last updated by author(s): Nov 23, 2020

Reporting Summary

Nature Research wishes to improve the reproducibility of the work that we publish. This form provides structure for consistency and transparency in reporting. For further information on Nature Research policies, see Authors & Referees and the Editorial Policy Checklist.

Statistics

For all statistical analyses, confirm that the following items are present in the figure legend, table legend, main text, or Methods section.

n/a	Confirmed	
☐	☒	The exact sample size (*n*) for each experimental group/condition, given as a discrete number and unit of measurement
☐	☒	A statement on whether measurements were taken from distinct samples or whether the same sample was measured repeatedly
☐	☒	The statistical test(s) used AND whether they are one- or two-sided<br>*Only common tests should be described solely by name; describe more complex techniques in the Methods section.*
☒	☐	A description of all covariates tested
☐	☒	A description of any assumptions or corrections, such as tests of normality and adjustment for multiple comparisons
☐	☒	A full description of the statistical parameters including central tendency (e.g. means) or other basic estimates (e.g. regression coefficient) AND variation (e.g. standard deviation) or associated estimates of uncertainty (e.g. confidence intervals)
☐	☒	For null hypothesis testing, the test statistic (e.g. *F*, *t*, *r*) with confidence intervals, effect sizes, degrees of freedom and *P* value noted<br>*Give P values as exact values whenever suitable.*
☐	☒	For Bayesian analysis, information on the choice of priors and Markov chain Monte Carlo settings
☒	☐	For hierarchical and complex designs, identification of the appropriate level for tests and full reporting of outcomes
☒	☐	Estimates of effect sizes (e.g. Cohen's *d*, Pearson's *r*), indicating how they were calculated

Our web collection on statistics for biologists contains articles on many of the points above.

Software and code

Policy information about availability of computer code

Data collection	NA
Data analysis	CHIP-seq and ATAC-seq data analysis<br>To generate the epigenomic annotations FASTQ files were obtained from Sequence Read Archive (SRA). Technical replicates were merged and Bowtie2(v2.4.1) was used for alignment for both single and paired-end files. FASTQC (v0.11.8) was used for quality control of the files. Resulting SAM files were filtered by MAPQ score and duplicates were removed using samtools(v1.9). MACS2 (v2.1.0) was used to call peaks for ATAC-seq and ChIP-seq files. Samtools mpileup function was used to quantify the number of reads that align to each allele.<br><br>Stratification into promoter/enhancer regions, stratification of ATAC-seq regions and partitioning heritability<br>We used HOMER(v4.11) annotatePeaks.pl function to obtain the distance of the peaks from transcription start sites. We used bed map(v2.4.36) to find overlapping regions. HOMER findMotifsGenome.pl function was used to identify motifs that are enriched in ATAC-seq regions associated with active enhancer profiles. HOMER annotatePeaks.pl and findMotifsGenome.pl functions were used to identify ATAC-seq regions that are positive for the motif of interest. Position weight matrices from HOMER were used. We used LD Score regression (v1.0.0) to quantify to quantify the enrichment of AD risk alleles in functional annotations.

Colocalization and Summary data-based Mendelian Randomization (SMR) analyses
Colocalization analyses were performed using coloc.abf function in coloc package (v3.2.1) in R (v3.5.3). To identify putative causal associations between epigenetic activity at active enhancers, gene expression and disease risk, we used SMR with default parameters. To identify the associations between chromatin QTLs (hQTLs) and gene expression, we set hQTLs as exposure and gene expression as outcome.

GCTA-Cojo conditoinal analyses
We used GCTA-COJO(v1.91.7beta) to conduct conditional analyses using IGAP GWAS summary statistics data and ADGC individual-level genotype data as a reference panel. Allele frequencies were estimated with ADGC using plink (v1.9). To conduct the conditional analysis we ran COJO with default parameters.

Prioritization of candidate causal variants
For each locus we constructed LD blocks using the CLQ algorithm for block partitioning within the BigLD package (no version listed on github). We then filtered for variants that reside in active enhancers in monocytes, macrophages and/or microglia (with the exception of the SPPL2A locus, since these variants likely regulate a distal enhancer as reported in Figure 3c). We also conducted a motif disruption/ creation analysis on these variants and selected the variants that are predicted to strongly disrupt or create binding sites of transcription factors that are expressed in myeloid cells (TPM≥1). We then screened the remaining variants for eQTLs in monocytes and macrophages from the Cardiogenics and Fairfax studies. We also used PAINTOR (v3.1) to conduct Bayesian fine-mapping in MS4A, ZYX and BIN1 loci. PAINTOR is a Bayesian fine-mapping method that leverages functional annotations through an Empirical Bayes prior. The input files for PAINTOR_v3.1 were prepared as described on the PAINTOR website and ADGC GWAS summary statistics along with individual-level genotype data were used for fine-mapping.The reprocessed epigenomic annotations were used to quantify enrichment at each locus. Quantification of annotation enrichment and fine-mapping were performed as described in the companion website.To classify the annotations as enriched or not, we computed the relative probability for a SNP to be causal given that it resides in the annotation as described in the companion website. We deemed the annotation to be significant if the relative probability of a SNP to be causal given that it is in the annotation was greater than 1. Once candidate causal variants were selected through both approaches, we conducted conditional analyses to make sure that they do indeed tag the majority of the GWAS signal in the locus.

Motif disruption analyses
To obtain evidence of SNPs that break the motif of a given transcription factor, we used motifbreakr, which is available as an R package. We used HOCOMOCO to screen for motifs and a P-value of significance of 5*10-5 as previously advised by the authors of the package.

For manuscripts utilizing custom algorithms or software that are central to the research but not yet described in published literature, software must be made available to editors/reviewers. We strongly encourage code deposition in a community repository (e.g. GitHub). See the Nature Research guidelines for submitting code & software for further information.

Data

Policy information about availability of data

All manuscripts must include a data availability statement. This statement should provide the following information, where applicable:

- Accession codes, unique identifiers, or web links for publicly available datasets
- A list of figures that have associated raw data
- A description of any restrictions on data availability

The following studies obtained from GEO were used for the analyses presented in this paper:GSE29611, GSE85245, GSE100380, GSE66594. Data generated in this study are available through accession number GSE164315. DbGAP accession study number for the human microglia dataset is phs001373.vl.pl. The genotype and phenotype data from ADGC are available under phs000372.vl.pl dbGAP study accession number. IGAP data can be found here:http://web.pasteur-lille.fr/en/recherche/u744/igap/igap_download.php. Blueprint eQTL data can be found here:https://www.blueprint-epigenome.eu/. The Cardiogenics dataset can be requested on EGA using accession number EGAS00001000411. DbGAP accession study number for the STARNET eQTL dataset is phs001203.v1.p1. Summary statistics for Fairfax eQTL data can be obtained from ArrayExpress using accession number E-MTAB-2232.

Field-specific reporting

Please select the one below that is the best fit for your research. If you are not sure, read the appropriate sections before making your selection.

☒ Life sciences ☐ Behavioural & social sciences ☐ Ecological, evolutionary & environmental sciences

For a reference copy of the document with all sections, see nature.com/documents/nr-reporting-summary-flat.pdf

Life sciences study design

All studies must disclose on these points even when the disclosure is negative.

Sample size	Sample sizes for: the GWAS (n= 74,046), hQTL(n=172) and eQTL(STARNET n=470, Fairfax n=432,Cardiogenics(mono/macro)n=849/684). These sample sizes were sufficient to detect genome-wide associations signals in AD risk loci and were hence sufficient for our analyses. We generated 3 iPSC-derived microglia lines for variant validation and 4 lines for RNA-seq analysis.
Data exclusions	No data were excluded.
Replication	The associations identified with the monocyte eQTL dataset from the Cardiogenics study were replicated in an independent monocyte eQTL dataset from the Fairfax study. Similarly, two independent macrophages eQTL datasets were used to replicate the findings, Cardiogenics and STARNET. Hence, we ran our analyses once in each monocyte and macrophage datasets, successfully replicating a large portion of AD risk genes within the same cell types.
Randomization	Because of the nature of the analyses we utilized (colocalization, SMR), we did not require random sampling, hence, randomization is not applicable.

Blinding	The only experiments where we tested the difference between groups were experiments where we tested the effect of the MS4A variants on open chromatin and expression. In these experiments, we did not have the knowledge of the genotype prior to testing. In ICC experiments, groups were not compared against each other , but rather microglial marker expression was confirmed.

Reporting for specific materials, systems and methods

We require information from authors about some types of materials, experimental systems and methods used in many studies. Here, indicate whether each material, system or method listed is relevant to your study. If you are not sure if a list item applies to your research, read the appropriate section before selecting a response.

Materials & experimental systems

n/a	Involved in the study
☐	☒ Antibodies
☐	☒ Eukaryotic cell lines
☒	☐ Palaeontology
☒	☐ Animals and other organisms
☒	☐ Human research participants
☒	☐ Clinical data

Methods

n/a	Involved in the study
☒	☐ ChIP-seq
☒	☐ Flow cytometry
☒	☐ MRI-based neuroimaging

Antibodies

Antibodies used

anti-TREM2 (R&D, AF1828), anti-P2RY12 (Sigma, HPA014518), anti-PU.1 (Cell Signaling, 2266) and anti-CX3CR1 (Bio-Rad, AHP1589). Secondary antibodies used were 1:300 Alexa donkey 488 and 568 anti-rabbit (Catalog # A-11011), mouse (Catalog # A28175), or chicken (Life Technologies,Catalog # A-11041). DAPI (4',6-diamidino-2-phenylindole, 0.5 μg/ml,Invitrogen, Cat# D1306) was used to visualize nuclei.

Validation

anti-TREM2 antibody: Some relevant citations provided by R&D Systems are below:
1) Zhong, L., Xu, Y., Zhuo, R., Wang, T., Wang, K., Huang, R., ... & Chen, K. (2019). Soluble TREM2 ameliorates pathological phenotypes by modulating microglial functions in an Alzheimer's disease model. Nature communications, 10(1), 1365.

2) Garcia-Reitboeck, P., Phillips, A., Piers, T. M., Villegas-Llerena, C., Butler, M., Mallach, A., ... & Neumann, H. (2018). Human induced pluripotent stem cell-derived microglia-like cells harboring TREM2 missense mutations show specific deficits in phagocytosis. Cell reports, 24(9), 2300-2311.

3) Jiang, H., Si, Y., Li, Z., Huang, X., Chen, S., Zheng, Y., ... & Xiong, H. (2016). TREM-2 promotes acquired cholesteatoma-induced bone destruction by modulating TLR4 signaling pathway and osteoclasts activation. Scientific reports, 6, 38761.

anti-P2RY12 antibody was developed and validated by Human Protein Atlas (HPA). Their validation procedures can be found here:https://www.proteinatlas.org/about/antibody+validation.

anti-PU.1 antibody: Some relevant citations provided by Cell Signaling are below:
1) Vargas, T. R., Cai, Z., Shen, Y., Dosset, M., Benoit-Lizon, I., Martin, T., ... & Apetoh, L. (2017). Selective degradation of PU. 1 during autophagy represses the differentiation and antitumour activity of TH 9 cells. Nature communications, 8(1), 559.

2) Huang, K. L., Marcora, E., Pimenova, A. A., Di Narzo, A. F., Kapoor, M., Jin, S. C., ... & Chouraki, V. (2017). A common haplotype lowers PU. 1 expression in myeloid cells and delays onset of Alzheimer's disease. Nature neuroscience, 20(8), 1052.

3) Abud, E. M., Ramirez, R. N., Martinez, E. S., Healy, L. M., Nguyen, C. H., Newman, S. A., ... & Caraway, C. A. (2017). iPSC-derived human microglia-like cells to study neurological diseases. Neuron, 94(2), 278-293.

anti-CX3CR1: relevant citations provided below
1) Julia, T. C. W., Liang, S. A., Qian, L., Pipalia, N. H., Chao, M. J., Shi, Y., ... & Holtzman, D. M. (2019). Cholesterol and matrisome pathways dysregulated in human APOE ε4 glia. bioRxiv, 713362.

Eukaryotic cell lines

Policy information about cell lines

Cell line source(s)	The samples were collected at the Alzheimer's disease research centers at University of California Irvine (UCI) and Washington University in St. Louis. The lines were reprogrammed into iPSCs at UCI and Mount Sinai.
Authentication	Sendai virus was used for reprogramming. The lines had normal and stable karyotypes. Pluripotency was assessed for OCT4, NANOG, TRA1-60, and TRA1-80.
Mycoplasma contamination	The cell lines were determined to be negative for micoplasma.
Commonly misidentified lines (See ICLAC register)	No commonly misidentified cell lines were used in this study.
